# Effect of volatile *versus* total intravenous anaesthesia on circulating tumour cells after pancreatic adenocarcinoma resection: multicentre randomized clinical trial

**DOI:** 10.1093/bjs/znad357

**Published:** 2023-11-14

**Authors:** Martin Schläpfer, Erik Schadde, Julia Braun, Christopher Soll, Stefan Breitenstein, Markus Weber, Stefan Gutknecht, Michael T Ganter, Miodrag Filipovic, Beatrice Beck-Schimmer

**Affiliations:** Institute of Anaesthesiology, University Hospital Zurich, University of Zurich, Zurich, Switzerland; Institute of Physiology, University of Zurich, Zurich, Switzerland; Institute of Physiology, University of Zurich, Zurich, Switzerland; Department of Surgery, Rush University Medical Centre, Chicago, Illinois, USA; Department of Surgery, Cantonal Hospital Winterthur, Winterthur, Switzerland; Departments of Epidemiology and Biostatistics, Epidemiology, Biostatistics and Prevention Institute, University of Zurich, Zurich, Switzerland; Department of Surgery, Cantonal Hospital Winterthur, Winterthur, Switzerland; Department of Surgery, Cantonal Hospital Winterthur, Winterthur, Switzerland; Department of Surgery, Triemli Hospital Zurich, Zurich, Switzerland; Department of Surgery, Triemli Hospital Zurich, Zurich, Switzerland; Institute of Anaesthesiology, Cantonal Hospital Winterthur, Winterthur, Switzerland; Department for Anaesthesiology, Intensive, Rescue and Pain Medicine, Cantonal Hospital St Gallen, St Gallen, Switzerland; Institute of Anaesthesiology, University Hospital Zurich, University of Zurich, Zurich, Switzerland; Institute of Physiology, University of Zurich, Zurich, Switzerland

## Introduction

Several retrospective studies have postulated that overall and recurrence-free survival decreases with use of volatile anaesthetics compared with intravenous agents for general anaesthesia in prostate^1^, oesophageal^2^, gastric^3^, and colorectal cancer^4^ resections. However, the level of evidence is low because well designed and properly performed RCTs are lacking. Moreover, the potential bias of retrospective data sets is impossible to eliminate by statistical methods.

Pancreatic adenocarcinoma is the fourth leading cause of cancer-related death in the USA^5^ and has a very high recurrence rates among the gastrointestinal cancers. In a curative setting, resectable pancreatic adenocarcinoma is removed by pancreatoduodenectomy (Whipple or Whipple–Kausch procedure) or distal pancreatectomy, both of which are complex surgical procedures requiring general anaesthesia.

Circulating tumour cells are released from the primary tumour, break into blood vessels, and colonize specific organs after systemic distribution, where they may set colonies and grow out as a distant metastasis^6^. A high circulating tumour cell count correlates well with advanced disease stage in pancreatic cancer^7^, and the presence of circulating tumour cells has been identified as a negative predictor of survival of patients with pancreatic adenocarcinoma^8^ as well as with shorter recurrence-free survival^9^.

As RCTs with an endpoint such as clinical outcome require a large number of subjects, a pilot was designed aiming at a biomarker such as circulating tumour cells.

## Methods

The trial was designed as a multicentre, parallel-group double-blinded RCT. Patients were assigned randomly to either desflurane or a propofol anaesthesia. Inclusion criteria were: age 18–85 years, ASA physical classification grade I–III, resectable pancreatic adenocarcinoma, primary surgery with the intent of complete tumour resection without neoadjuvant therapy, and signed informed consent. The study took place in three tertiary care hospitals in Eastern Switzerland (Cantonal Hospital St Gallen, Cantonal Hospital Winterthur, and Triemli Hospital Zurich). The full study protocol can be found in the *supplementary material*.

### Outcomes

Circulating tumour cells were measured in full blood (7.5 ml) before resection under general anaesthesia (T0), 3 days after the resection (T1), at 7 days (T2), and 1–3 months (T3), 6 months (T4), and 12 months (T5) after resection. The measurement at T3 had to be done before the initiation of chemotherapy. The primary outcome was the peak level of circulating tumour cells on day 3 or 7 after surgery (T1 or T2). To obtain the most precise counts of circulating tumour cells, the measurements were performed using the CellSearch® (Menarini Silocon Biosystems, Huntington Valley, PA) system, which remains the standard system for measuring circulating tumour cells to date^10^.

Secondary outcomes were: the kinetics of circulating tumour cells up to 1 year after surgery, time to tumour recurrence, and overall survival. The time outcomes were obtained from patient charts. This study was not powered for analysis of tumour recurrence and overall survival.

### Statistical analysis

The peak circulating tumour cell value was first compared between the two groups using the Mann–Whitney *U* test, and afterwards using a negative binomial regression model that accounted for overdispersion. It was adjusted for baseline circulating tumour cells and the presence of microvascular invasion, lymph node (N) status, and residual tumour after resection (R1/R2). Mixed negative binomial models with a random intercept for each individual were used to analyse the course of the circulating tumour cell counts over time, adjusting for the same variables as before. The log rank test and Cox regression models were used to compare the time to recurrence and overall survival between the two groups. They were adjusted for the presence of microvascular invasion, N status, and residual tumour after resection.

The impact of missing data was assessed using multiple imputation using chained equations with 50 imputed data sets. For all results, 95 per cent confidence intervals were calculated and a two-sided level of significance of 5 per cent was used.

## Results

### Patient flow and baseline characteristics

The study flow chart is shown in *Fig. S1*. Demographic and clinical characteristics at baseline were similar in the two groups (*Table S1*). Tumour characteristics are described in *Table S1*, and intraoperative and postoperative data in *Table S2*.

### Primary outcome

The circulating tumour cell counts of all patients over the entire study interval are shown in *Fig. S2*.

**Fig. 1:**
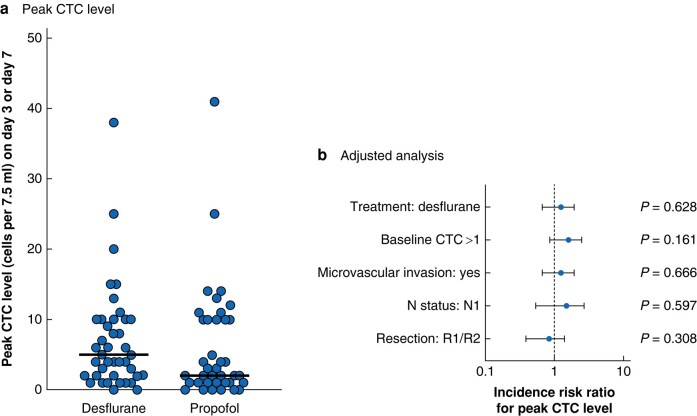
Analysis of maximum circulating tumour cell levels **a** Distribution of maximum circulating tumour cell (CTC) levels in the two treatment groups; bold lines represent median values. **b** Results of negative binomial regression models considering the confounders group allocation, baseline CTC more than 1 per 7.5 ml blood, microvascular invasion, lymph node (N) status, and resection (R1/R2). Incidence risk ratios are shown with 95 per cent confidence intervals.

There was no significant difference in the peak levels of circulating tumour cells on postoperative day 3 or 7 between the desflurane and the propofol groups (*P* = 0.18, Mann–Whitney *U* test) (*Fig. 1a*). The median value was 5 (i.q.r. 2–10) in 7.5 ml blood in the desflurane group and 2 (1–10) in 7.5 ml blood in the propofol group. The treatment was found to have no effect on peak circulating tumour cell counts after adjusting for baseline circulating tumour cell levels, the presence of microvascular invasion, lymph node status, and residual tumour after resection (R1/R2) (*Fig. 1b*). Multiple imputation did not show any relevant differences in comparison with these results.

### Secondary outcomes

The dynamics of circulating tumour cells from before chemotherapy until year 1 after surgery remained unaffected by the treatment with desflurane in a basic negative binomial model. Neither desflurane (incidence risk ratio (IRR) 0.900, 95 per cent c.i. 0.56 to 1.47; *P* = 0.684), nor baseline circulating tumour cells (IRR 1.02, 1.00 to 1.04; *P* = 0.135), nor time measured in days (IRR 1.00, 1.00 to 1.00; *P* = 0.397) had any impact on the circulating tumour cell count.

**Fig. 2:**
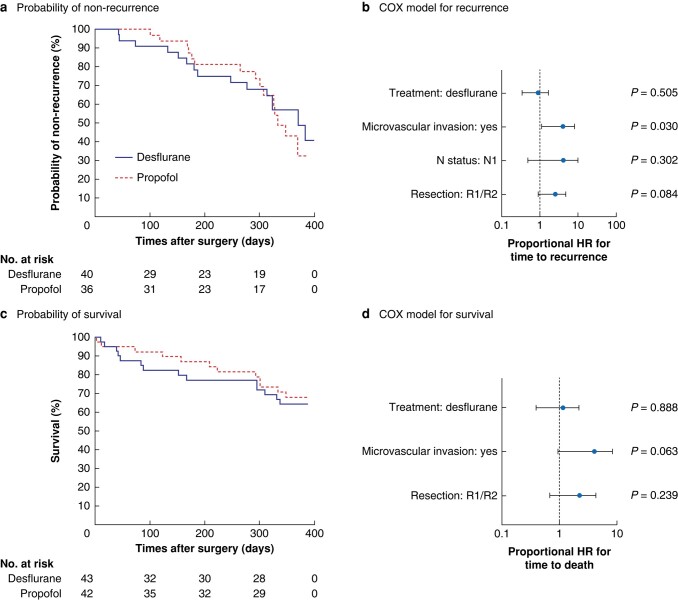
Analysis of recurrence and survival **a** Kaplan–Meier plot showing probability of non-recurrence in desflurance and propofol groups (*P* = 0.589, log rank test). **b** Cox regression model for time to recurrence, including the confounders group allocation, microvascular invasion, lymph node (N) status, and resection (R1/R2). **c** Kaplan–Meier plot showing probability of survival in desflurance and propofol groups (*P* = 0.806, log rank test). **d** Cox regression model for time to death, considering the confounding factors treatment, microvascular invasion, and resection (R1/R2). HRs are shown with 95 per cent confidence intervals.

The time to recurrence was similar in both groups (*P* = 0.589) (*Fig. 2a*), resulting in a disease-free survival rate 1 year after surgery of 57 per cent in the desflurane and 40 per cent in the propofol group. A Cox proportional hazards model showed a significant association between the presence of microvascular invasion and time of recurrence (HR 3.01, 95 per cent c.i. 1.11 to 8.14; *P* = 0.030), whereas there was no evidence of an effect of the type of anaesthesia (HR 0.77, 0.35 to 1.67; *P* = 0.505), N1 status (HR 2.20, 0.49 to 9.86; *P* = 0.302), and completeness of resection (R1/R2) (HR 2.08, 0.91 to 4.78; *P* = 0.084) (*Fig. 2b*).

There was no difference in overall survival between the two anaesthesia groups (*Fig. 2c*), with rates of 66 per cent in the desflurane and 68 per cent in the propofol group at 1 year (*P* = 0.806). An adjusted Cox proportional hazards model did not show an association between type of anaesthesia and overall survival (HR 0.94, 0.40 to 2.22; *P* = 0.888) (*Fig. 2d*).

## Discussion

To the authors’ knowledge, this is the first randomized trial to compare a volatile anaesthetic with a totally intravenous anaesthetic in patients undergoing resection of pancreatic adenocarcinoma. The study design, namely the assessment of both biological markers (circulating tumour cells) and clinical events (recurrence and overall survival over 12 months of follow-up), allows a less biased investigation of the effect of two different general anaesthesia regimens on the postoperative course of patients with pancreatic adenocarcinoma.

This study has several limitations. Neither recurrence nor overall survival was assessed as a primary endpoint. A second limitation is that the power calculation was not accurate owing to the limited availability of data at the time of designing the trial. Finally, an imbalance in patients with non-curative resection—4 per cent in the desflurane and 21 per cent in the propofol group—has to be highlighted (*Table S1*). Although a randomized approach was chosen, this imbalance could have been due to the small number of patients included.

In summary, this RCT found no evidence that desflurane or propofol anaesthesia had an impact on postoperative circulating tumour cell levels.

## Supplementary Material

znad357_Supplementary_Data

## Data Availability

Data are available upon request to the corresponding or first authors.
