# Supplementary material for: Effect of volatile versus total intravenous anaesthesia on circulating tumour cells after pancreatic adenocarcinoma resection: multicentre randomized clinical trial
Source: Br J Surg. 2023 Nov 14;111(1):znad357. doi: 10.1093/bjs/znad357 (PMC10771133; doi:10.1093/bjs/znad357)
Supplement: znad357_Supplementary_Data [file znad357_supplementary_data.docx]

**Effect of volatile *vs.* total intravenous anesthesia on circulating tumor cells after pancreatic adenocarcinoma resection.**

**A multicenter randomized controlled trial.**

Martin Schläpfer^1,2^, Erik Schadde^2,3,4^, Julia Braun^5^, Christopher Soll^4^, Stefan Breitenstein^4^, Markus Weber*^6^*, Stefan Gutknecht*^6^*, Michael T Ganter*^7^*, Miodrag Filipovic^8^, and Beatrice Beck-Schimmer^1,2^

^1^ Institute of Anesthesiology, University Hospital Zurich, University of Zurich, Zurich, Switzerland

^2^ Institute of Physiology, University of Zurich, Zurich, Switzerland

^3^ Department of Surgery, Rush University Medical Center, Chicago, Illinois

^4^ Department of Surgery, Cantonal Hospital Winterthur, Canton of Zurich, Switzerland

^5^ Departments of Epidemiology and Biostatistics, Epidemiology, Biostatistics and Prevention Institute, University of Zurich, Zurich, Switzerland

## 6 Department of Surgery, Triemli Hospital Zurich, Zurich, Switzerland

^7^ Institute of Anesthesiology, Cantonal Hospital Winterthur, Canton of Zurich, Switzerland

^8^ Department for Anesthesiology, Intensive, Rescue and Pain medicine, Cantonal Hospital St. Gallen, Canton of St. Gallen, Switzerland

**^#^ Corresponding author** (work primarily attributed to the University of Zurich)

Beatrice Beck-Schimmer, M.D., Institute of Anesthesiology, University Hospital and University of Zurich, Raemistrasse 100, CH-8091 Zurich; Phone: +41 44 635 50 35; email: [beatrice.beckschimmer@uzh.ch](mailto:beatrice.beckschimmer@uzh.ch)

**Funding statement**

This work was supported by a grant from the Swiss National Science Foundation, SNSF, Bern, Switzerland (grant Nr. 32-160283) (BBS), Swiss Society for Anesthesiology and Resuscitation (SGAR), Bern, Switzerland (BBS), Baxter Healthcare Corporation, Deerfield, IL (BBS), a private foundation located in Switzerland (BBS), the (BBS) and the Vontobel Foundation, Zurich, Switzerland. The funding organizations had no role in the design and conduct of the study; nor in the collection, management, analysis and interpretation of the data; nor in the preparation, review or approval of the manuscript; nor in the decision to submit the manuscript for publication.

**Category**

Short Reports (randomized controlled trial)

**Prior presentations**

Data were presented at the virtual Annual Meeting of the Swiss Society for Anesthesiology and Resuscitation (SGAR) on October 29 and 30, 2020.

**Conflict of interest statement**

BBS has a patent 04/10/14 – 20140100278: Injectable formulation for treatment and protection of patients having an inflammatory reaction or an ischemia-reperfusion event; M. Urner, L.K. Limbach, I.K. Herrmann, W.J. Stark, B. Beck-Schimmer, applied as Patent Cooperation Treaty (PCT) (internationally), July 2009, as well as a patent application on bioconjugates of antibodies and functionalized magnetic nanoparticles. BBS and MS received Sedana Medical AB, Danderyd, Sweden, grant money as collaborators of a large multicenter study ‘Inhaled Sedation in COVID-19-related Acute respiratory distress syndrome (ISCA): an international research data study in the recent context of widespread disease resulting from the 2019 (SARS-CoV2) coronavirus pandemics (COVID-19), not related to this topic. MS and BBS have received grant money from Roche Diagnostics International, Rotkreuz, Switzerland, for a clinical trial not related to this topic.

**Data availability statement**

Data are available upon request (corresponding

**Supplementary Materials - Index**

| **Supplementary Material Manuscript** |  |
| --- | --- |
| Material  Results  Discussion  References | *Page 3*  *Page 8*  *Page 10*  *Page 11* |
| **Supplementary Appendixes** |  |
| Protocol submitted to the ethic’s committee | *Page 12* |
| **Supplementary Figures and Tables** |  |
| Figures | *Page 54* |
| Tables | *Page 60* |

**Supplementary Methods**

*Trial design*

Local ethics committees approved the study (approval number: ZH-2016-00448). Written informed consent was obtained from all subjects or a legal surrogate. The study was registered on ClinicalTrials.gov (NCT02335151) and is reported according to the consolidated standards of reporting trials (CONSORT) checklist ^1^. All centers had monitoring that was independent of the group of investigators.

*Randomization*

Participants were randomly assigned to intravenous propofol or volatile desflurane anesthesia at a 1:1 allocation ratio shortly before entering the operating theatre. A secure internet-based system ([www.randomizer.at](http://www.randomizer.at)) was used for randomization.

The randomization was pre-stratified for the center and the type of surgery (laparoscopy vs. open resection) to prevent confounding due to these factors and was performed by investigators at the respective centers.

*Blinding*

The patients, the biostatistician, and the laboratory staff determining circulating tumor cells were all blinded. The primary endpoint (circulating tumor cells), tumor recurrence and overall survival were taken from patient charts that were filled in by their treating doctors and nurses. None of these had anything to do with the treatment allocation and were thus fully blinded. However, the group assignment was known to the anesthesiologists and the surgeons involved in the procedures per the pragmatic limitations of blinding.

*Participants*

All patients with resectable pancreatic ductal adenocarcinoma undergoing primary pancreatic surgery were consecutively screened in the preoperative anesthesiology clinic. The inclusion criteria were age 18 - 85 years, American Society of Anesthesiology physical classification (ASA) I – III, resectable pancreatic adenocarcinoma (i.e. no imaging evidence of borderline resectability features as defined by the national comprehensive cancer network, NCCN, guidelines; no locally advanced disease), primary surgery with the intention of complete tumor resection without prior neoadjuvant therapy and signed informed consent. The exclusion criteria were metastatic disease, history of previous pancreatic resection, locally advanced or borderline resectable tumors, need for neoadjuvant chemotherapy, chronic opioid use, suspected or known intolerance to propofol, soy or egg proteins, and malignant hyperthermia. Pregnant and breast-feeding women were excluded, as were patients enrolled in other clinical trials between 30 days prior to pancreatic adenocarcinoma surgery and 30 days after completion of this trial. Withdrawal of consent led to the exclusion of the patient from analysis due to ethical reasons. Following the intention-to-treat approach, those patients who could not be resected due to intraoperative findings remained included.

*Anesthesia and surgery interventions*

Upon arrival in the operating room, patients received a peripheral intravenous line, standard monitoring with a 5-lead electrocardiogram, pulse oximetry, and invasive or non-invasive blood pressure monitoring. Thoracic epidural anesthesia was performed in case of preoperative consent to combined anesthesia. After the installation of a bi-spectral index (BIS) monitoring (Covidien, USA) and preoxygenation, anesthesia was induced in both groups with intravenously applied fentanyl 2-3mcg kg^-1^ and thiopental 3-6mg kg^-1^. Either atracurium 0.5mg kg^-1^ or rocuronium 0.6mg kg^-1^ was used for neuromuscular blockade. In the case of rapid sequence induction, the dose of rocuronium was adapted to 0.9mg kg^-1^. After intubation, patients were ventilated with an inspiratory oxygen fraction of 0.4. Anesthesia was then maintained with either propofol or with desflurane according to group allocation by randomization. The dose was titrated to a target BIS of 40-60. If clinically necessary, fentanyl boluses of 1-2mcg kg^-1^ and/or continuous remifentanil infusion up to 20mcg kg^-1^ h were applied. Neuromuscular blockade was monitored by train of four (TOF) stimulation of the ulnar nerve and was sustained with 5-10mg rocuronium or atracurium boluses in case of a TOF response of ≥2. To minimize a potentially confounding effect of local anesthetics on circulating tumor cell count and oncological outcomes, local anesthetics were limited to thoracic epidural anesthesia. Postoperative analgesia was provided with acetaminophen and non-steroidal anti-inflammatory drugs. Patients received postoperative opioids in the presence of pain of visual analogue scale (VAS) ≥ 4. Postoperative nausea and vomiting (PONV) prophylaxis and therapy were performed according to departmental guidelines.

According to the principles of surgical oncology, the goal was a complete tumor resection. Whipple-Kausch procedures, distal pancreatectomies, central pancreatectomies, or total pancreatectomy, all en bloc with locoregional lymph node resection, were performed according to the localization of the tumor. If indicated, portal vein reconstructions were performed. Adjuvant chemotherapy was given to all patients who tolerated it medically, according to the MDT decision.

*Outcomes*

Circulating tumor cells were determined using the CellSearch system that enumerates circulating tumor cells of epithelial origin (CD45-, EpCAM+, and cytokeratins 8, 18+, and/or 19+) in whole blood. Fifteen ml of whole blood was collected into CellSave preservative tubes and transported (at room temperature) to the Experimental Anesthesiology Laboratory at the University Hospital Zurich for subsequent analysis within 96 hours of blood collection. All measurements were made according to CellSearch manufacturer specifications and analyzed by two independent and specially trained research staff members as previously described ^2^. The number of circulating tumor cells refers to 7.5ml blood.

*Sample size*

The sample size was determined per a Poisson regression model, and the peak levels of circulating tumor cells were compared at postoperative days 3 and 7 for the desflurane and the propofol group with 80% power and a level of significance of 0.05. A peak circulating tumor cell rate of 6 in 7.5ml blood (which under the Poisson assumption corresponds to a variance of 6 and an according standard deviation of 2.45) was assumed in the control group, and a response rate ratio of 0.77 was considered relevant. This led to a total sample size of 86 patients (including 3 dropouts per group).

In the first protocol version a peak value of 10 circulating tumor cells in 7.5ml blood was assumed. The sample size was 56 patients including 6 drop-outs. After an interim analysis with the first 10 patients a peak value of 6 circulating tumor cells was found, leading to a correction of the sample size adding additional 30 patients (86 patients including 3 drop-outs per group) (protocol version 1.3, 2017-07-14 in the **Supplementary Appendixes**, again approved by the local ethics committees).

*Statistical methods*

The statistical analysis plan was approved by the ethics committee and the authors before analyses began.

Data that is normally distributed are presented as mean and standard deviation (SD) and data that is not normally distributed is presented as median and interquartile range (IQR). Categorical data are summarized as numbers (n) and proportions of the total (%). The data was analyzed using the intention-to-treat approach.

**Supplementary Results**

*Patient flow*

Between October 2016 and September 2018, 144 patients were screened for eligibility across the three Swiss study centers. Eleven patients were excluded. After providing written informed consent, 85 patients were randomly assigned to propofol or desflurane and were included in the intention-to-treat analysis (**Figure S1**).

The circulating tumor cell measurements for the completeness of the primary endpoints (circulating tumor cells on days 3 and 7) were 93% and 90% for the desflurane group and 98% and 77% for the propofol group, respectively. Following the intention-to-treat principle, the maximum circulating tumor cell value was based on one single measurement if one of the two was missing. At baseline, 4 analyses are missing due to technical or logistical problems. On day 3, 12 analyses could not be performed due to technical or logistical problems; one analysis is missing because the patient withdrew his consent, and one patient died.

*Baseline characteristics*

  Demographic and clinical characteristics at baseline were similar in the two groups (**Table S1**). The median age was 71 years (63-79 IQR) in the desflurane and 71 years (65-75 IQR) in the propofol group, and patients had a median BMI of 25 kg m^-2^ (22-29 IQR) and 26 kg m^-2^ (23-27 IQR), respectively. More than 60% belonged to the ASA group III. Tumor stages, as well as involvement of lymph nodes assessed according to TNM UICC classification version 7, were comparable in the two allocation groups.

Tumor characteristics are indicated in **Table S1**, intra- and postoperative data in **Table S2**.

*Primary outcome*

The circulating tumor cell counts of all patients over the entire study duration are shown in **Figure S2**.

*Subgroup analysis*

A per protocol analysis was performed including only the subgroup of patients who had surgery with the intention of curative resection. Of the 85 patients included in the study, 74 underwent such resection. Due to a locally advanced or metastatic tumor situation, resection was not carried out for 2 patients in the desflurane group and 9 in the propofol group (**Table S2**). In the “as-treated” cohort, a negative binomial model also found that there was no relationship between the type of anesthesia (IRR 1.14, 95% CI: from 0.66 to 1.96, p=0.63), the baseline circulating tumor cell levels (IRR 1.40, 95% CI: from 0.80 to 2.44, p=0.22), the presence of microvascular invasion (IRR 1.07, 95% CI: from 0.60 to 1.86, p=0.81), and the N1-stage (IRR 1.18, 95% CI: from 0.50 to 2.57, p=0.68) with the peak levels of circulating tumor cells at day 3 or 7 (**Figure S3**).

In the per-protocol analysis, both time to recurrence and probability of overall survival were comparable in the two anesthesia groups. The type of anesthesia did not reach significance with regard to the probability of disease-free survival one year after surgery (HR 0.83, 95% CI: from 0.38 to 1.82, p=0.64) (**Figure S4A and S4B**). Overall survival at one year did not differ between the desflurane group and the propofol group (HR 0.91, 95% CI: from 0.39 to 2.14, p=0.84) (**Figure S5A and S5B**).

**Supplementary Discussion**

An additional interesting finding of this study is the high circulating tumor cell count, both in the immediate postoperative phase (on day 3 and on day 7) and in the 1-3-month period *prior to initiation* of chemotherapy, a period in which all of the local tumor has been presumably resected in most patients. The number of circulating tumor cells (median, IQR) (in 7.5ml blood) remained elevated in both groups from a preoperative cell count of 1 (0-4) to postoperative day 3 with 2 circulating tumor cells (0-6) and day 7 with 2 circulating tumor cells (0-6), highlighting that circulating tumor cells were not fully eliminated through resection of the tumor. At 3 months, i.e., before initiation of chemotherapy, circulating tumor cell levels remained elevated and unchanged compared to preoperative levels with 1 circulating tumor cell (0-2) (**Figure S6**). Gemenetzis et al. showed that a median of 9 circulating tumor cells /ml in peripheral blood (IQR 5-12) in the preoperative phase changed to a median of 2 (IQR 1-3) 4-6 days after pancreatic cancer resection ^3^, corroborating our findings of persistence of circulating tumor cells after resection with curative intent. We were able to show that the number of circulating tumor cells also consolidates at 6 and 12 months at a lower level, albeit with the circulating tumor cells still being detectable at 1 circulating tumor cell (0-2) at 6 months and 1 circulating tumor cell (0-2) at 12 months (both in 7.5ml blood), but without a difference between the groups. This supports the observation that pancreatic adenocarcinoma is a systemic disease that is due to either the continued presence of micrometastases that release circulating tumor cells or to the general lack of clearance of circulating tumor cells from the bloodstream in pancreatic adenocarcinoma after resection with curative intent. This finding is underlined by the fact that neither the baseline circulating tumor cells nor the microvascular invasion nor the N stage had any impact on the peak levels of circulating tumor cells, recurrence (except the microvascular invasion) and overall survival.

**References**

1. Schulz KF, Altman DG, Moher D, Group C. CONSORT 2010 statement: updated guidelines for reporting parallel group randomised trials. *BMJ* 2010;**340**: c332.

2. Hovaguimian F, Braun J, Z'Graggen B R, Schlapfer M, Dumrese C, Ewald C, Dedes KJ, Fink D, Rolli U, Seeberger M, Tausch C, Papassotiropoulos B, Puhan MA, Beck-Schimmer B. Anesthesia and Circulating Tumor Cells in Primary Breast Cancer Patients: A Randomized Controlled Trial. *Anesthesiology* 2020;**133**(3): 548-558.

3. Gemenetzis G, Groot VP, Yu J, Ding D, Teinor JA, Javed AA, Wood LD, Burkhart RA, Cameron JL, Makary MA, Weiss MJ, He J, Wolfgang CL. Circulating Tumor Cells Dynamics in Pancreatic Adenocarcinoma Correlate With Disease Status: Results of the Prospective CLUSTER Study. *Ann Surg* 2018;**268**(3): 408-420.

**Appendix 1: Protocol submitted to the ethic’s committee**

Study synopsis 10

Abbreviations 15

Study schedule 16

1. STUDY ADMINISTRATIVE STRUCTURE 17

1.1. Sponsor 17

1.2. Coordinating Investigator 17

1.3. Principal Investigators 17

1.4. Statistician ("Biostatistician") 17

1.5. Collaborators 18

1.6. Laboratory 18

1.7. Monitoring institution 18

1.8. Data Safety Monitoring Committee 18

1.9. Any other relevant committee, person, organisation, institution 19

2. ETHICAL AND REGULATORY ASPECTS 19

2.1. Study registration 19

2.2. Categorisation of study 19

2.3. Competent Ethics Committee (CEC) 19

2.4. Competent Authorities (CA) 19

2.5. Ethical conduct of the study 19

2.6. Declaration of interest 20

2.7. Participant information and informed consent 20

2.8. Participant privacy and confidentiality 20

2.9. Early termination of the study 20

2.10. Protocol amendments 21

3. Introduction 21

3.1. Background and rationale 21

3.2. Investigational product and indication 23

3.3. Preclinical evidence 24

3.4. Clinical evidence to date 24

3.5. Dose rationale: Rationale for the intended purpose in study 24

3.6. Explanation for choice of comparator 24

3.7. Risks / Benefits 25

3.8. Justification of choice of study population 25

4. STUDY OBJECTIVES 25

4.1. Overall objective 25

4.2. Primary objective 25

4.3. Secondary objectives 26

4.4. Safety objectives 26

5. STUDY OUTCOMES 26

5.1. Primary outcome 26

5.2. Secondary outcomes 26

5.3. Other outcomes of interest 26

5.4. Safety outcomes 26

6. STUDY DESIGN 27

6.1. General study design and justification of design 27

6.2. Methods of minimising bias 28

6.2.1. Randomization 28

6.2.2. Blinding procedures 28

6.2.3. Other methods of minimising bias 28

6.3. Unblinding procedures (Code break) 28

7. STUDY POPULATION 28

7.1. Eligibility criteria 28

7.2. Recruitment and screening 29

7.3. Assignment to study groups 29

7.4. Criteria for withdrawal / discontinuation of participants 29

8. STUDY INTERVENTION 29

8.1. Identity of investigational oroducts (treatment) 29

8.1.1. Packaging, Labelling and Supply (re-supply) 29

8.2. Administration of experimental and control interventions 30

8.2.1. Experimental Intervention 30

8.2.2. Control Intervention 30

8.3. Dose modifications 30

8.4. Compliance with study intervention 30

8.5. Data collection and follow-up for withdrawn participants 30

8.6. Trial specific preventive measures 30

8.7. Concomitant Interventions (treatments) 30

8.8. Study drug accountability 31

8.9. Return or destruction of study drug / medical device 31

9. STUDY ASSESSMENTS 31

9.1. Study flow chart of study procedures and assessments 31

9.2. Assessments of outcomes 32

9.2.1. Assessment of primary outcome 32

9.2.2. Assessment of secondary outcomes 32

9.2.3. Assessment of safety outcomes 32

9.2.4. Assessments in participants who prematurely stop the study 32

9.3. Procedures at each visit 33

9.3.1. Visit 1 (screening visit, preoperative phase) 34

9.3.2. Visit 2 (day of surgery – before induction of general anesthesia,) 34

9.3.3. Visit 3 (day 3 after surgery) 34

9.3.4. Visit 4 (day 7 after surgery) 34

9.3.5. Visit 5 and 6 and 7 (pre-chemotherapy, 6- and 12-month follow-up) 34

10. SAFETY 35

10.1. Definitions 35

10.2. Recording of Serious Adverse Events 35

10.3. Assessment of Serious Adverse Events 36

10.4. Reporting of Serious Adverse Events 36

10.5. Follow up of (Serious) Adverse Events 37

11. STATISTICAL METHODS 37

11.1. Hypothesis 37

11.2. Determination of sample size 37

11.3. Statistical criteria of termination of trial 38

11.4. Planned analyses 38

11.4.1. Datasets to be analysed, analysis populations 38

11.4.2. Primary Analysis 38

11.4.3. Secondary Analyses 38

11.4.4. Interim analyses 38

11.4.5. Safety analysis 38

11.4.6. Deviation(s) from the original statistical plan 38

11.5. Handling of missing data and drop-outs 39

12. Eligibility of the Project Site(s) 39

13. QUALITY ASSURANCE AND CONTROL 39

13.1. Data handling and record keeping / archiving 39

13.1.1. Case Report Forms 39

13.1.2 Randomization number Record keeping / archiving 40

13.2. Data management 40

13.2.1. Data management system 40

13.2.2. Data security, access and back-up 41

13.2.3. Analysis and archiving 41

13.2.4. Electronic and central data validation 41

13.3. Monitoring 41

13.4. Audits and inspections 41

13.5. Confidentiality, data protection 41

13.6. Storage of biological material and related health data 42

14. PUBLICATION AND DISSEMINATION POLICY 42

15. FUNDING AND SUPPORT 42

15.1. Funding 42

15.2. Other support 42

16. INSURANCE 42

17. REFERENCES 43

18. APPENDICES 44

Study synopsis

| Sponsor | Prof. Dr. med. Beatrice Beck Schimmer |
| --- | --- |
| Study Title: | Resectable pancreatic adenocarcinoma–  Does the type of anesthesia have an impact on circulating tumor cells  A randomized, double blind, controlled trial |
| Short Title / Study ID: | CTC pancreatic adenocarcinoma |
| Protocol Version and Date: | Version 1.3, 14.07.2017 |
| Trial Registration | SNCTP: 01892  KEK: 2016 – 00448  Clinicaltrials.gov: NCT 02335151 |
| Study category and Rationale | Risk category A |
| Clinical Phase: | Clinical Phase category IV,  Desflurane (Suprane®) is authorized in Switzerland and its use is in accordance with the prescribing information (indication, application). |
| Background and Rationale: | Several clinical trials have shown that circulating tumor cells (CTC) in cancer patients have prognostic value for tumor recurrence. Little information is available for the perioperative phase when patients undergo cancer surgery. It is known that anesthetics have an immunomodulatory effect. We hypothesize that the type of anesthetic (volatile versus intravenous) may impact determine the level of CTC in the perioperative phase and thereby influence tumor recurrence. |
| Objective(s): | **Primary objective:**  To assess the effects of the general anesthetic desflurane on peak CTC levels in the postoperative phase compared to propofol in patients with resectable pancreatic adenocarcinoma undergoing pancreatic resection.  **Secondary objective:**  To compare effects of desflurane and propofol on CTC kinetics postoperatively and during follow-up, on local and systemic tumor recurrence as well as on overall survival in patients with pancreatic cancer after curative surgery. |
| Outcome(s): | **Primary outcome:**  Peak levels of postoperative CTC after curative surgery in patients with adenocarcinoma of the pancreas.  **Secondary outcomes:**  1) CTC levels from day 0 to day 7 (repeated measurements) after curative  surgery in patients with adenocarcinoma of the pancreas. 2) CTC at time  point before first chemotherapy cycle (approximately 1-3 months  postoperative (pre-chemotherapy), 6 and 12 months after curative surgery. 3)  Time to tumor recurrence (local or distant recurrence) as assessed from the  date of resection to the date of first recurrence. Patients who are free of  tumor recurrence will be censored at the time of death or at the last follow-up  (1 year). 4) Overall survival defined as the period from surgery to the date of  death from any cause or the last contact if the patient was alive. |
| Study design: | Prospective, randomized, multicenter, double blind, controlled trial |
| Inclusion / Exclusion criteria: | Key Inclusion Criteria:  - Age 18 to 85  - ASA I-III  - Resectable pancreatic adenocarcinoma  - Primary surgery with intention of complete tumor resection  - No neoadjuvant treatment  - Written informed consent  Key Exclusion Criteria:  - Metastatic disease  - History of previous pancreatic resection  - Preoperative chemotherapy  - Chronic opioid use  - Suspected or known intolerance by history to propofol, soya or egg proteins  - Suspected or known intolerance by history to volatile anaesthetics (malignant hyperthermia)  - Pregnancy  - Breast feeding  - Enrollment in any other clinical trial during the course of this trial, 30 days prior to its beginning or 30 days after its completion |
| Measurements and procedures: | Patients will be randomized to anesthesia with either propofol or desflurane. CTC will be determined at the following time points: 1) Preoperative time zero before surgery (T0) 2) 3 days after surgery (T1) 3) 7 days after surgery (T2) 4) At follow-up after 1-3 (pre-chemotherapy), 6 and 12 months (T3-T5). |
| Study Product / Intervention: | Desflurane will be used in its inhalational form via an endotracheal tube for maintenance of anesthesia. The dose and regimen will be adjusted to keep an adequate depth of anesthesia, which will be monitored by bispectral index (BIS, value of 40-60). |
| Control Intervention: | Propofol will be administered intravenously using target-controlled infusion TCI) or total intravenous anesthesia, TIVA, aiming at an adequate and comparable depth of anesthesia (BIS value of 40-60). |
| Number of Participants with Rationale: | 86 patients will participate, 43 in each study group (including 3 drop-outs per group). |
| Study Duration: | Duration of subject participation: up to 1 year after surgery  Duration of study: Recruitment period of 2 years, follow-up period of 1 year = 3 years in total |
| Study Schedule: | 06/2016 of First Participant-In (planned)  05/2019 of Last-Participant-Out (planned) |

| Investigator(s): | Prof. Dr med. Stefan Breitenstein  Department of Surgery  Kantonsspital Winterthur  Brauerstr. 15  8401 Winterthur  Email: stefan.breitenstein@ksw.ch  Phone: +41 52 266 24 02  Fax: +41 52 266 24 54  Dr. med. Erik Schadde  Department of Surgery  Kantonsspital Winterthur  Brauerstr. 15  8401 Winterthur  Email: erik.schadde@uzh.ch  Phone: +41 78 952 73 68  PD Dr. med. Michael Ganter  Department of Anesthesiology  Kantonsspital Winterthur  Brauerstr. 15  8401 Winterthur  Email: Michael.ganter@ksw.ch  Phone: +41 52 266 27 92  Fax: +41 52 266 45 18  Prof Dr. med. Miodrag Filipovic  Kantonsspital St. Gallen  Division of Anesthesiology; Intensiv Care, Rescue and Pain Medicine  Rorschacherstr. 95  9007 St. Gallen  Email: miodrag.filipovic@kssg.ch  Phone: +41 71 494 15 05  Fax: +41 71 494 28 89  Prof. Dr. med. Markus Weber  Stadtspital Triemli  Departement of Surgery  Birmensdorferstr. 497  8063 Zurich  Email: markus.weber@triemli.zuerich.ch  Phone: +41 44 416 43 01  Fax: +41 44 416 43 20  Prof. Dr. med. Andreas Zollinger  Stadtspital Triemli  Institute of Anesthesiology and Intensiv care  Birmensdorferstr. 497  8063 Zurich  Email: andreas.zollinger@triemli.zuerich.ch  Phone: +41 44 416 52 02  Fax: +41 44 416 52 10 |
| --- | --- |
| Investigator(s): | Dr. med. Martin Schläpfer  University Hospital Zürich  Institute of Anesthesiology  Rämistr. 100  8091 Zurich  Email: martin.schlaepfer@usz.ch  Phone: +41 44 255 11 11  Fax: +41 44 255 44 09 |
| Study Center(s): | Kantonsspital Winterthur, Switzerland Kantonsspital St. Gallen, Switzerland Stadtspital Triemli, Switzerland |
| Statistical Considerations: | Patient will be randomized 1:1 in the two arms. We consider relative reduction in peak CTC count of at least 20% in patients with the intervention as relevant compared to patients with the control intervention. A poisson regression of a dependent variable on a binomial distributed independent variable using a sample of 86 patients would achieve 80% power at the 0.05 significance level for a 2-sided test to detect a response rate ratio of at least 0.77 for intervention group compared with the control group count if the postoperative peak rate was 6 and the mean exposure time was 1. For the power calculation, we assumed 3 drop outs per group. |
| GCP Statement: | This study will be conducted in compliance with the protocol, the current version of the Declaration of Helsinki, the ICH-GCP or ISO EN 14155 (as far as applicable) as well as all national legal and regulatory requirements. |

Abbreviations

Provide a list of abbreviations used on the protocol - to be completed

| AE | Adverse Event |
| --- | --- |
| BIS  CA | Bispectral index  Competent Authority (e.g. Swissmedic) |
| CEC | Competent Ethics Committee |
| ClinO | Ordinance on Clinical Trials in Human Research |
| CRF  eCRF | Case Report Form  Electronic Case Report Form |
| CTC | Circulating Tumor Cells |
| CTCAE | Common terminology criteria for adverse events |
| DSUR | Development safety update report |
| ECOG  ERAS  ESDO  ESMO  GCP | Eastern Cooperative Oncology Group  Enhanced Recovery After Surgery  European Society of Digestive Oncology  European Society of Medical Oncology  Good Clinical Practice |
| IB | Investigator’s Brochure |
| Ho | Null hypothesis |
| H1 | Alternative hypothesis |
| HFG | Humanforschungsgesetz (Law on human research) |
| HMG | Heilmittelgesetz |
| HRA | Federal Act on Research involving Human Beings |
| IMP | Investigational Medicinal Product |
| IIT | Investigator-Initiated Trial |
| ISO | International Organisation for Standardisation |
| ITT | Intention to Treat |
| PI | Principal Investigator |
| PONV | Postoperative nausea an vomiting |
| SDV | Source Data Verification |
| SOP | Standard Operating Procedure |
| SPC | Summary of Product Characteristics |
| SUSAR | Suspected Unexpected Serious Adverse Reaction |
| TMF | Trial Master File |
| TCI | Target-Controlled Infusion |
| TIVA | Total Intravenous Anesthesia |
| VAS | Visual Analog Scale for pain |

WHO World Health Organization

Study schedule

**Table 1**

| Study Periods | Screening | Treatment | Post OP | | Follow-up | | |
| --- | --- | --- | --- | --- | --- | --- | --- |
|  |  |  |  |  |  |  |  |
| Visit | 1 | 2 | 3 | 4 | 5 | 6 | 7 |
|  |  |  |  |  |  |  |  |
| Time ( day, month) | -7d | 0 | 0+3d | 0 + 7d | pre-chemo | +6m | +12m |
| Patient Information and Informed Consent | X |  |  |  |  |  |  |
| Demographics | X |  |  |  |  |  |  |
| Medical History | X |  |  |  |  |  |  |
| In- /Exclusion Criteria | X | X | X | X |  |  |  |
| Physical Examination | X |  |  |  | X | X | X |
| Vital Signs | X | X | X | X |  |  |  |
| CTC (primary variable) |  | X | X | X | X | X | X |
| Pregnancy Test | X |  |  |  |  |  |  |
| Randomization |  | X |  |  |  |  |  |
| Perioperative Data |  | X |  |  |  |  |  |
| Administer Study Medication |  | X |  |  |  |  |  |
| Secondary Variables |  |  |  |  | X | X | X |
| Concomitant Therapy (Radiotherapy, Chemotherapy) |  |  |  |  | X | X | X |
| Laboratory Tumor Marker | X |  |  |  | X | X | X |
| Adverse Events |  | X | X | X |  |  |  |

| Visit 1: | Up to -7 days prior to surgery |
| --- | --- |
| Visit 2: | Before induction of anesthesia |
| Visit 3: | 3 days after surgery |
| Visit 4: | 7 days after surgery |
| Visit 5, 6, 7: | Follow up, pre-chemo (1-3 months), 6 and 12 months after surgery |

# STUDY ADMINISTRATIVE STRUCTURE

## 1.1. Sponsor

Name: Prof. Dr. med. Beatrice Beck Schimmer

Address: Universitätsspital Zürich

Institut für Anästhesiologie

Rämistrasse 100, 8091 Zürich

Email: beatrice.beck@usz.ch

Phone: +41 44 255 20 32

Fax: +41 44 255 44 09

## 1.2. Coordinating Investigator

Name. Prof. Dr. med. Breitenstein Stefan

Address: Kantonsspital Winterthur

Department of Surgery

Brauerstrasse 15

8401 Winterthur

Email: stefan.breitenstein@ksw.ch

Phone: +41 52 266 27 92

## 1.3. Principal Investigators

Name. Prof. Dr. med. Breitenstein Stefan

Address: Kantonsspital Winterthur

Department of Surgery

Brauerstrasse 15

8401 Winterthur

Email: stefan.breitenstein@ksw.ch

Phone: +41 52 266 27 92

Fax: +41 52 266 45 18

Name. Prof. Dr. med. Filipovic Miodrag

Address: Kantonsspital St. Gallen

Division of Anesthesiology, Intensive Care, Rescue and Pain Medicine

Rorschacherstr. 95

9007 St. Gallen

Email: miodrag.filipovic@kssg.ch

Phone: +41 71 494 15 05

Fax: +41 71 494 28 89

Name. Prof. Dr. med. Weber Markus

Address: Stadtspital Triemli

Department of Surgery

Birmensdorfestr. 497

8063 Zürich

Email: markus.weber@triemli.zuerich.ch

Phone: +41 44 466 22 02

Fax: +41 44 466 26 01

## 1.4. Statistician ("Biostatistician")

Prof. Dr. med. Puhan Milo and Dr. Braun Julia

Epidemiology, Biostatistics and Prevention Institute

Hirschengraben 84

8001 Zurich

Email: miloalan.puhan@ uzh.ch

Phone: +41 44 634 46 10

## 1.5. Collaborators

Prof Dr. med. Bruno Schmied

Kantonsspital St. Gallen

Department of Surgery

Rorschacherstr. 95

9007 St. Gallen

Email: bruno.schmied@kssg.ch

Phone: +41 71 494 13 12

Fax: +41 71 494 28 86

Prof. Dr. med. Pless Miklos

Address: Kantonsspital Winterthur

Rorschacherstr. 95

9007 St. Gallen

Email: miklos.pless@ksw.ch

Phone: +41 52 266 36 40

Fax: +41 52 266 45 20

Prof. Dr. med. Güller Ulrich

Address: Kantonsspital St. Gallen

Rorschacherstr. 95

9007 St. Gallen

Email: Ulrich.gueller@kssg.ch

Phone: +41 71 494 26 38

Fax: +41 71 494 63 25

Dr. med. John Bonvini

Ospedale regionale di Lugano – Civico e Italiano

Institute of Anesthesiology

Via Tesserete 46

6900 Lugano

## 1.6. Laboratory

CTC isolation and enumeration will be performed by using CellSearch®

Laboratory Group Prof. Dr. med Beck Schimmer

University Hospital Zurich

D LAB 29

Sternwartstr.14

8091 Zurich

## 1.7. Monitoring institution

The trial will be monitored by the Sponsor.

## 1.8. Data Safety Monitoring Committee

A data safety monitoring board (DSMB) is not needed because the investigational products are used in the daily routine in anesthesia departments.

##

## 1.9. Any other relevant committee, person, organisation, institution

N/A

# ETHICAL AND REGULATORY ASPECTS

Before the study will be conducted, the protocol, the proposed patient information and consent form as well as other study-specific documents are submitted to a properly constituted Competent Ethics Committee (CEC). Any amendment to the protocol must as well be approved by CEC.

The decision of the CEC concerning the conduct of the study will be made in writing to the Sponsor before commencement of this study. The clinical study can only begin once approval from all required authorities has been received. Any additional requirements imposed by the authorities shall be implemented.

## 2.1. Study registration

SNTCP: 01892

KEK: 2016-00448

Clinicaltrials.gov: NCT02335151

## 2.2. Categorisation of study

The risk category of this study is category A.

The IMP is authorized in Switzerland and its use is in accordance with the prescribing information.

## 2.3. Competent Ethics Committee (CEC)

Approval from the appropriate constituted CEC is sought for each study site in the clinical trial. The reporting duties and allowed time frame are respected. No substantial amendments are made to the protocol without prior Sponsor and CEC approval, except where necessary to eliminate apparent immediate hazards to study participants.

Premature study end or interruption of the study is reported within 15 days. The regular end of the study is reported to the CEC within 90 days, the final study report shall be submitted within one year after study end. Amendments are reported according to chapter 2.10.

## 2.4. Competent Authorities (CA)

No approval from Swissmedic is necessary for this category A clinical trial.

## 2.5. Ethical conduct of the study

The study will be carried out in accordance to the protocol and with principles enunciated in the current version of the Declaration of Helsinki, the guidelines of Good Clinical Practice (GCP) issued by ICH, in case of medical device: the European Directive on medical devices 93/42/EEC and the ISO Norm 14155 and ISO 14971, the Swiss Law and Swiss regulatory authority’s requirements. The CEC and regulatory authorities will receive annual safety and interim reports and be informed about study stop/end in agreement with local requirements.

## 2.6. Declaration of interest

No conflict of interests has to be declared.

## 2.7. Participant information and informed consent

The investigators will explain to each participant the nature of the study, its purpose, the procedures involved, the expected duration, the potential risks and benefits and any discomfort it may entail. Each participant will be informed that the participation in the study is voluntary and that he/she may withdraw from the study at any time and that withdrawal of consent will not affect his/her subsequent medical treatment.

The participant must be informed that his/her medical records may be examined by authorised individuals other than their treating physician.

All participants for the study will be provided a participant information sheet and a consent form describing the study and providing sufficient information for participant to make an informed decision about their participation in the study. Enough time needs to be given to the participant to decide whether to participate or not. Please specify the time frame given.

The patient information sheet and the consent form will be submitted to the CEC to be reviewed and approved. The formal consent of a participant, using the approved consent form, must be obtained before the participant is submitted to any study procedure.

The participant should read and consider the statement before signing and dating the informed consent form, and should be given a copy of the signed document. The consent form must also be signed and dated by the investigator (or his designee) and it will be retained as part of the study records.

## 2.8. Participant privacy and confidentiality

The investigator affirms and upholds the principle of the participant's right to privacy and that they shall comply with applicable privacy laws. Especially, anonymity of the participants shall be guaranteed when presenting the data at scientific meetings or publishing them in scientific journals.

Individual subject medical information obtained as a result of this study is considered confidential and disclosure to third parties is prohibited. Subject confidentiality will be further ensured by utilising subject identification code numbers (coding) to correspond to treatment data in the computer files. The coding key will be kept in a locked location only accessible to the principal investigator at the respective study center. Safety of this information will be in the individual responsibility of the principal investigator.

Such medical information may be given to the participant’s personal physician or to other appropriate medical personnel responsible for the participant’s welfare, if the patient has given his/her written consent to do so.

For data verification purposes, authorised representatives of the Sponsor a competent authority or an ethics committee may require direct access to the coding key and to parts of the medical records relevant to the study, including participants’ medical history.

## 2.9. Early termination of the study

The Sponsor may terminate the study prematurely according to certain circumstances, for example:

- ethical concerns,
- insufficient participant recruitment,
- when the safety of the participants is doubtful or at risk, respectively,
- alterations in accepted clinical practice that make the continuation of a clinical trial unwise,
- early evidence of benefit or harm of the experimental intervention

## 2.10. Protocol amendments

Only the sponsor is allowed to amend important protocol modifications such as changes to eligibility criteria, outcomes and/or analyses to CEC.

Substantial amendments are clinically only implemented after approval of the CEC.

Under emergency circumstances, deviations from the protocol to protect the rights, safety and well-being of human subjects may proceed without prior approval of the sponsor and the CEC. These deviations are within the responsibility of the investigators. Such deviations shall be documented and reported to the sponsor and the CEC as soon as possible.

All non-substantial amendments are communicated to the CEC within the Annual Safety Report (ASR).

# Introduction

## 3.1. Background and rationale

Pancreatic adenocarcinoma and circulating tumor cells

Adenocarcinoma of the pancreas represents the most common type of pancreatic cancer. According to the American Cancer Society 46’420 new cases of pancreatic cancer are estimated in the United States with 39’590 deaths per year (American Cancer Society; www.cancer.com). Among cancer-related deaths in the United States, pancreatic cancer ranks as the fourth most common ^1^. The global mortality rate is 98% ^2^. Due to an advanced local disease or metastatic disease at the time point of diagnosis, only 10-20% of the patients are initially resectable. These patients have a 5-year survival of approximately 10-24% after complete resection^3, 4^, reflecting the aggressive and lethal nature of this cancer. This problem has remained unresolved so far. Recurrence rate is 80% after curative resection^4^.

Although their significance is controversial, the detection of circulating tumor cells (CTC) in the peripheral blood of cancer patients has received increasing interest within the past decade. Adenocarcinoma of the breast is a tumor entity with well-characterized CTC. Patients with, breast cancer, have a shorter progression-free survival and a shorter overall survival they test “positive” for CTC (i.e. ≥5 CTC/7.5ml blood)^5, 6^. A recently published meta-analysis pooling more than 6’800 patients investigated the prognostic value of CTC in breast cancer ^7^. The presence of CTC was associated with a significantly increased risk of disease recurrence (HR 2.86, 95%CI 2.19-3.75) and with significantly higher mortality rates (HR 2.78, 95%CI 2.22-3.48). This analysis also provided evidence that the presence of CTC was significantly associated with poorer prognosis in both early-stage and metastatic breast cancer, regardless of the detection method (CellSearch assay or reverse-transcriptase polymerase chain reaction (RT-PCR) approach).

For pancreatic cancer little data is available with regard to CTC detection. The following table summarizes results of CTC studies for pancreatic adenocarcinoma with an enrollment of more than 15 patients^8^:

**Table 1**


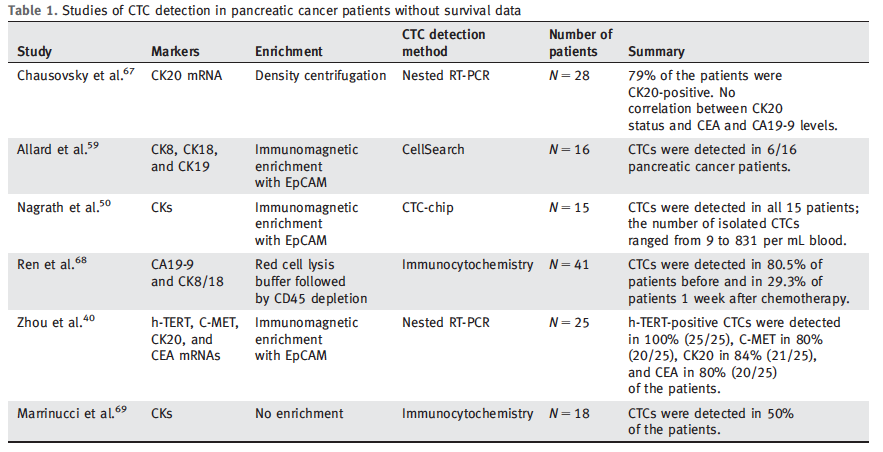


In the study of Allard et al. CTC detection of pancreatic cancer was performed using CellSearch®^9^, which is the same device we are planning to use as well. Out of 16 patients with pancreatic cancer 4 had more than 2 CTC/7.5 ml, 6 more than 1 CTC/7.5ml.

Whether CTC evolve into metastatic disease and/or local recurrence is certainly a complex process, in which immunity plays a key role^10^. Despite these increasing efforts have been made to identify likely factors leading to the presence of CTC in blood, since each step of ‘tumor dissemination’ represents a potential target for new cancer therapy ^10^. Tumor manipulation, for instance, has been thought to participate in CTC spreading. A recent study pointed out, that CTC after tumor removal increased abruptly in 5 out of 6 patients (83%). Interestingly, no increase was seen after the no-touch isolation of pancreaticoduodenectomy (n=6) ^11^.

Anesthesia and immunity

An increasing body of evidence suggests that perioperative stress induces immunosuppression in cancer patients ^12^. Along with surgery, pain, hypothermia or blood transfusions, anesthetic drugs are considered to play a role in the postoperative immune dysfunction^13, 14^. Additionally, recent findings suggest that the type of anesthesia may have an impact on recurrence of malignancies after surgical removal. ^15^.

A recent *in vitro* study showed that migration of colonic cancer cells through a Matrigel, simulating extracellular matrix, was significantly impaired in the presence of the volatile anesthetic sevoflurane or desflurane. This effect was explained by decreased metalloproteinase-9 levels after volatile anesthesia ^16^.


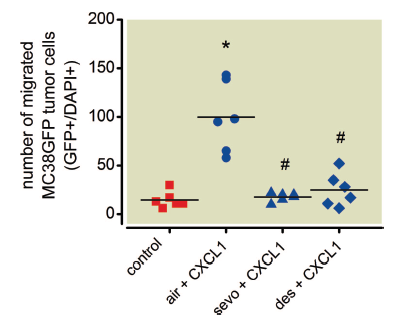


**Figure 1:** Neutrophils were preconditioned, stimulated with CXC-ligand 1 (CXCL1) and co-incubated with colon cancer cells (MC38). Cells were allowed to transmigrate through a Matrigel. Transmigrated cancer cells were counted ^16^.

CTC isolation and enumeration

The CellSearch system consists of a semi-automated system (CellPrep) for the preparation of the sample, and it is used with the CellSearch Epithelial Cell Kit. The CellPrep system enriches the sample for cells expressing EpCAM with antibody-coated ferrous particles, and it labels the cell nucleus with the fluorescent nucleic acid dye 4,2-diamidino-2-phenylindole dihydrochloride. Fluorescence labeled monoclonal antibodies specific for leukocytes (CD45-allophycocyan) and cytokeratins (CK 8, 18, 19-phycoerythrin) are used to distinguish epithelial cells from leukocytes. The identification and enumeration of CTC are performed using the CellSpotter Analyzer, a semi-automated fluorescence-based microscopy system that permits computer-generated reconstruction of cellular images. CTC are defined as nucleated cells that lack CD45, but express cytokeratin. Technical details of the CellSearch and CellSpotter systems, including accuracy, precision, linearity, and reproducibility, have been described elsewhere ^17^.

**Research question:** With the proposed study we aim to compare the impact of the anesthetics desflurane and propofol on the kinetic of CTC after pancreatic adenocarcinoma resection. Pancreatic adenocarcinoma is an epithelial cancer with one of highest recurrence rates. We hypothesize that desflurane decrease CTC when compared to propofol. Additionally, our small cohort serves to explore if this reduction in CTC correlates with a better tumor outcome.

## 3.2. Investigational product and indication

Suprane® (desflurane)


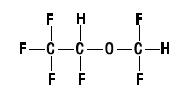


(±)1,2,2,2-tetrafluoroethyl difluoromethyl ether

Desflurane is a colorless, non-flammable; volatile liquid (below 22.8°C) administered via vaporizer for general inhalation anesthetic.

The administration of general anesthesia must be individualized based on the patient’s response. The dose and regimen will be adjusted to keep an adequate depth of anesthesia (BIS 40-60).

Administration will be limited to the length of the surgical procedure.

Because of its little solubility in blood and tissues its effects are of rapid onset. Recovery of consciousness is usually achieved within 5 - 15 minutes after discontinuation of application.

see appendix N° 1 (Fachinformation)

##

## 3.3. Preclinical evidence

There is no preclinical evidence exists about a beneficial effect of desflurane on tumor progression

##

## 3.4. Clinical evidence to date

In 2014 we started a clinical trial focusing on the kinetic of CTC in breast cancer patients after removal of the primary tumor. Patients are being randomized for intravenous *vs.* volatile anesthesia. CTC are determined using the CellSearch® system, which is highly feasible. The study is ongoing, and results have not been analyzed yet. Importantly, CTC are detectable in these patients and are highly variable (Study KEK 2013-0408; ClinicalTrials.gov NCT02005770).

Please see also 3.1. Background and rationale.

##

## 3.5. Dose rationale: Rationale for the intended purpose in study

The use of desflurane as a general anesthetic is nowadays well established, safe clinical practice throughout the anesthesiologist community. Dosage of desflurane is performed by the anesthesiologist, based on experience and clinical findings during any particular procedure. The dosage used refers to a normal concentration of desflurane, which is used to perform a general anesthesia.

The choice between the different regimens of anesthesia maintenance (volatile vs. intravenous) is mostly dictated by habits and local hospital practices.

## 3.6. Explanation for choice of comparator

Propofol is an intravenous hypnotic agent, poorly soluble in water, thus dissolved in a fat emulsion vehicle that contains soya bean oil, triglycerides, egg lecithin, glycerol, sodium oleate and water.

2,6-diisopropylphenol

Its hypnotic effect occurs rapidly (less than 60 seconds), but the effect duration is short, due to rapid redistribution and metabolism. It is a well-established drug, which has been successfully used for general anesthesia or sedation for more than 20 years.

Since the emergence of short acting intravenous anesthetics in the late 80’s, a large amount of literature about these novel intravenous drugs has been published to comparing them with conventional gas. The studies have focused on pharmacological effects, patient safety, quality process, patient satisfaction and organ protection. No consensus regarding the superiority of intravenous *vs*. volatile anesthesia has been reached so far, mainly because postoperative complications are multifactorial (surgery, preoperative comorbidities) and to single out the effect of the type of anesthesia alone requires large randomized studies. In the meantime, with the advent of novel volatile drugs like sevoflurane and desflurane the question of the superiority of volatile anesthesia has come to the forefront again.

Both desflurane and propofol may cause the following effects during general anesthesia:

- Cardiovascular: hypotension (fall in systemic resistance), bradycardia.
- Respiratory: reduced minute ventilation reduced respiratory threshold to hypoxia, increased tolerance to hypercapnia.
- Central nervous system: autoregulation maintained, decrease in cerebral blood flow and oxygen consumption.
- Propofol is known to decrease the incidence of postoperative nausea and vomiting in susceptible individuals.
- In soya- or egg-allergic patients, the administration of propofol can induce anaphylactic reactions.

## 3.7. Risks / Benefits

Each anesthesia bears a risk. Study patients have to undergo major surgery and therefore application of a general anesthesia is inevitable. Both anesthetics are used for this type of surgery. In clinical practice it is the choice of the anesthesiologist in charge, if a volatile or intravenous anesthesia is performed. Therefore no modifications outside of a normal anesthesia are foreseen (no off label use).

##

## 3.8. Justification of choice of study population

Patients with resectable adenocarcinoma of the pancreas undergoing both pancreaticoduodenectomy (Whipple-Kausch procedure) and pancreatic body and/or tail resection will be included. We chose this tumor entity because of its poor prognosis. In this disease, local or systemic tumor recurrence will be observed within the first year after resection. In this disease, one-year follow-up will be a meaningful endpoint.

# STUDY OBJECTIVES

## 4.1. Overall objective

This prospective, randomized, double blind, controlled study will be conducted over a 36-month period to investigate whether commonly used anesthetics (volatile *versus* intravenous) have an effect on the changes or occurrence of CTC in patients suffering from primary pancreatic cancer undergoing curative surgery. The specific research question is if there are changes of the CTC count in the postoperative phase in the desflurane group (intervention) compared to the propofol one (control). A secondary question is if these changes can indeed be correlated with a more favorable tumor outcome. In a small study like this, a correlation found may be meaningful. Due to its size, however, the study cannot rule out a correlation if the study’s findings are negative.

## 4.2. Primary objective

Primary objective is to compare the impact of desflurane and propofol on peak CTC levels (per 7.5ml blood, using the CellSearch® approach) in the postoperative phase (up to T3: day 7).

## 4.3. Secondary objectives

1) To compare CTC levels from day 0 to day 7 (repeated measurements) after curative surgery in patients with adenocarcinoma of the pancreas. 2) to compare the impact of desflurane and propofol on CTC at T3: 1-3 months, before start of first chemotherapy cycle, T4: 6 months and T5: 12 months after surgery. 3) to compare the impact of desflurane and propofol on time to local and systemic tumor recurrence as assessed from the date of resection to the date of first recurrence. 4) To compare the impact of desflurane and propofol on overall survival defined as the period from surgery to the date of death from any cause or the last contact if the patient was alive. 5) to assess the association of CTC count in the postoperative phase with recurrence and overall survival .

## 4.4. Safety objectives

In every study that randomizes a therapeutic intervention, however well established and accepted, safety is always an additional objective. Any SAE as described below is registered.

# STUDY OUTCOMES

## 5.1. Primary outcome

Primary endpoint will be the peak CTC levels after curative tumor removal of pancreatic adenocarcinoma.

## 5.2. Secondary outcomes

Secondary outcomes are defined as:

1) Kinetics of CTC after surgery up to day 7 (i.e. repeated measurements)

2) CTC at T3: 1-3 months (pre-chemotherapy, first cycle), T4: 6 months and T5: 12 months after surgery

3) Time (months) to tumor recurrence (local or systemic recurrence) as assessed from the date of resection to the date of first recurrence. Patients who are free of tumor recurrence will be censored at the time of death or at the last follow-up (1 year).

4) Overall survival (months) defined as the period from surgery to the date of death from any cause or the last contact if the patient was alive.

## 5.3. Other outcomes of interest

Not applicable

## 5.4. Safety outcomes

Study drugs will be used in accordance with the prescribing information (indication, application). Both anesthetics are approved by Swissmedic. There are no specific safety concerns because the study drugs are using within standard indications.

However, any SAE will be reported from signing the patient informed consent to discharge of hospital and the causal relationship of the anesthetic regimen to them will be examined in every case. Specific attention will be paid to the question if the randomization to one study arm as opposed to the choice of the anesthesiologist in charge played a role in the complication. Policies and procedures of reporting of complications are described in chapter 10.

# STUDY DESIGN

We will conduct a prospective, randomized, multicenter, double blind, controlled trial (RCT) with the primary endpoint CTC load in the early postoperative phase in patients with pancreatic adenocarcinoma after resection. CTC load in the late postoperative phase as well as clinical outcomes survival and recurrence are defined as secondary endpoints.

## 6.1. General study design and justification of design

Patients considered for the study will be screened for eligibility according to the inclusion and exclusion criteria at any time point prior to surgery. Written informed consent will be subsequently obtained by one of the investigators. A pregnancy test will be performed for all included premenopausal female patients, as is clinical routine prior to major abdominal surgery.

*Anesthesia*

On the day of surgery, patients will receive oral premedication according to local guidelines.

After preoxygenation, induction of anesthesia will be administrated in both groups similarly, using fentanyl 2-3 mcg/kg, thiopental 3-6 mg/kg and rocuronium 0.6 mg/kg or atracurium 0.5 mg/kg intravenously (i.v.).

Patients requiring a rapid sequence induction will receive rocuronium 0.9 mg/kg instead of 0.6 mg/kg.

In both groups, after induction, patients will be ventilated using an oxygen inspiratory fraction of ≥0.4.

Depth of hypnosis will be assessed by bispectral index (BIS) reflecting electrical activity in the brain with target BIS values of 40-60. The following anesthetics will be used:

1) desflurane in the “group desflurane”

2) propofol in the “group propofol”

In both groups, when deemed clinically necessary, analgesia will be supplemented by applying fentanyl 1-2 mcg/kg and / or continuous remifentanyl infusion up to 20 mcg/kg/h. Muscle relaxation will be monitored by train of four (TOF) stimulation of the ulnar nerve and supplemented with 5-10 mg rocuronium, or alternatively atracurium, after TOF response of 2 or more.

To minimize the potential confounding effect of local anesthetics on CTC counts and/or oncological outcomes, local anesthetics will be limited to epidural anesthesia performed following local guidelines. No systemic lidocaine infusion and surgical wound infiltration will be performed.

Following the commonly used Enhanced Recovery After Surgery (ERAS) protocol will be allowed.

Basic postoperative analgesia will be provided using paracetamol and non-steroidal anti-inflammatory drugs. Opioids, iv and/or sc will be available for patients with a Visual Analog Scale for Pain (VAS score) ≥ 4 according to local departmental guidelines. Postoperative nausea and vomiting (PONV) prophylaxis and treatment will be prescribed following departmental guidelines, ~~excluding steroids~~.

*Operation*

According to the principles of surgical oncology the goal of the surgical treatment of resectable adenocarcinoma of the pancreas is a macroscopically complete resection of the tumor (R0 or R1). The standard operation for tumors of the pancreatic head is the pancreaticoduodenectomy or Whipple-Kausch procedure, either in its pylorus-preserving or non-pylorus preserving variant, whereas tumors of the body or tail can be resected using a distal pancreatectomy, or, in rare cases, a central pancreatic resection In some patients with multilocular cancer or underlying cystic diseases of the pancreas, a total pancreatectomy may be required. A pancreatic resection includes a locoregional lymphadenectomy. Portal vein reconstruction may be indicated in cases of portal vein infiltration or as a routine depending on the judgment of the surgeon.

*Adjuvant Chemotherapy*

Adjuvant chemotherapy has proven advantageous in terms of prolonging overall survival. In accordance with the standards of medical oncology, patients of the present trial may receive adjuvant chemotherapy after pancreatic resection according to the decision of the tumor board of the respective institution.

## 6.2. Methods of minimising bias

### Randomization

Randomization will be done by a computer- generated anesthesia-assignment. (www.randomizer.at) This process should be performed as close as possible to the application of the study drugs. We will use randomization and stratification by center and surgical technique (laparoscopy/laparotomy). Also, the strict inclusion/exclusion criteria will already provide some control for important confounders.

### 6.2.2. Blinding procedures

Adequate blinding will be performed: Patients will not be informed about their group assignments, The lab staff performing the CTC detection will not have any access to the operation theatre or to the patient chart (= double blind trial). Due to the procedures involved in volatile versus intravenous anesthesia, group assignment cannot be entirely concealed for the anesthesiologists and surgeons involved with the procedure in the operating room (pragmatic limits of blinding).

### 6.2.3. Other methods of minimizing bias

NA

## 6.3. Unblinding procedures (Code break)

The attending anesthesiologist responsible for randomization and providing the anesthesia and collecting the first blood sample cannot *per se* be blinded, but will not participate in any data collection or analysis. The study research team collecting the data will not have access to patient allocation until the final analysis.

Due to the fact that the anesthesiologist in charge is not blinded, there is no need for an emergency code break.

# STUDY POPULATION

The study population consists of patients with adenocarcinoma of the pancreas (head, tail) undergoing primary surgery in a curative intent (head or tail, the latter also known as left resection).

## 7.1. Eligibility criteria

Participants fulfilling all of the following inclusion criteria are eligible for the study, for example:

- Age 18 to 85

- American Society of Anesthesiologists (ASA) physical status I-III

- Suspected or confirmed resectable pancreatic adenocarcinoma

- Primary surgery with intention of complete tumor resection

- No neoadjuvant treatment

- Written informed consent

The presence of any one of the following exclusion criteria will lead to exclusion of the participant

- Metastatic disease

- History of previous pancreatic resection

- Preoperative chemotherapy

- Chronic opioid use

- Suspected or known intolerance by history to propofol, soya or egg proteins

- Suspected or known intolerance by history to volatile anaesthetics (malignant hyperthermia)

- Pregnancy

- Breast feeding

- Enrollment in any other clinical trial during the course of this trial, 30 days prior to its beginning or 30 days after its completion

## 7.2. Recruitment and screening

Consecutive patients with pancreatic cancer evaluated by the groups of the surgical investigators will be screened for resectability and then for the other eligibility criteria. All patients will also be assessed by the anesthesiological investigators in the presurgical anesthesiology clinics. The attending anesthetist will inform patients about the study orally and in writing. Patients willing to participate will subsequently provide written informed consent. A pregnancy test will be performed in female patients in childbearing age.

Participation is on a strictly voluntary base. No compensation in any form will be granted to the subjects*.*

## 7.3. Assignment to study groups

See chapter 6.2.1.

Assignment into study group will be performed by randomization using [www.randomizer.at](http://www.randomizer.at) just before providing the study drugs.

## 7.4. Criteria for withdrawal / discontinuation of participants

Patients will be informed about the possibility to retract their participation to the study at any time. In case of withdrawal of consent, patients will be excluded from the study. Other than the withdrawal of consent scenario, patients will not be excluded in keeping with an intent-to-treat approach. In case of non-resectability on exploration or incomplete resection of the tumor (R2), patients will not be excluded from the analysis.

Investigators may withdraw a patient prior to expected completion of the study for safety reasons (for instance as the result of an adverse event (AE) with unacceptable consequence or risk for the patient). In both cases, for patient safety, a final medical examination will be performed. Notification of the discontinuation will be clearly documented on the patient’s case report form (CRF).

Since data collection and analysis will be performed following an intention-to-treat model, all consecutive patients will be analyzed according to the initial randomization group, regardless of the study drug administered. These patients will not be withdrawn from analysis.

# STUDY INTERVENTION

## 8.1. Identity of investigational oroducts (treatment)

**Desflurane (intervention group)** Suprane®, Baxter, 250ml bottle

**Propofol (control group)** Disoprivan® 1% (10mg/ml), AstraZeneca, 50ml bottle

### Packaging, Labelling and Supply (re-supply)

Both desflurane (Suprane®) and propofol (Disoprivan 1% (10mg/ml), AstraZeneca) are anesthetics used on a daily base in all study centers. The product will be taken out of stock in each affiliated center and will be labelled as follows:

FOR CLINICAL TRIAL USE ONLY

CTC_PANCREATIC ADENOCARCINOMA

Sponsor: Beck Schimmer Beatrice

Study ID:

Desflurane (Suprane®) OR Propofol (AstraZeneca)

Charge:

Expiry Date:

Storage: at room temperature (15-25°C), never over 30°C.

Patient ID:

- - 1. **Storage Conditions**

Study products will be stored in a storage facility accessible exclusively to members of the study group. The principal investigators will be responsible to make sure that enough medications are in storage to perform the trial. Regarding the volatile anesthesia, a vaporizer will be filled with desflurane and stored in the locker. This vaporizer will be used exclusively for study patients in each center and will be stored back in the locker at the end of the anesthesia.

## 8.2. Administration of experimental and control interventions

### 8.2.1. Experimental Intervention

Group desflurane (Suprane®): Anesthesia will be maintained with desflurane in >40% oxygen (inspiratory oxygen), adjusted to keep an adequate depth of anesthesia (BIS 40-60). Drug administration should be limited to the length of the surgical procedure.

### Control Intervention

Group propofol: Anesthesia will be maintained with a target-controlled infusion (TCI) or total intravenous infusion (TIVA) providing a continuous intravenous propofol dose guaranteeing an adequate anesthesia depth (BIS 40-60). Patients will be ventilated with >40% oxygen (inspiratory oxygen concentration). Drug administration should be limited to the length of the surgical procedure.

## 8.3. Dose modifications

In case of aggravated fluid (blood) loss patients in the propofol group might experience a more pronounced instability of the cardiovascular system than desflurane patients. It will be up to the decision of the responsible anesthesiologist to stop the propofol infusion and initiate desflurane anesthesia. Patients data will be analyzed in an intent-to treat fashion.

## 8.4. Compliance with study intervention

Because the attending anesthesiologist will provide the administration of study drugs, treatment compliance is not considered as a potential issue.

## 8.5. Data collection and follow-up for withdrawn participants

Patients unexpectedly meeting exclusion criteria upon internal monitoring will be followed during the study course and evaluated following intent-to-treat.

All data of included patients will be recorded in the CRF unless the patient specifically withdraws his consent. Withdrawn patients will undergo a final examination by the investigator before discharge.

## 8.6. Trial specific preventive measures

A pregnancy test will be performed in all women of childbearing age routinely prior to the procedure. Pregnant patients will not be included.

Patients being on any systemic immunosuppressive therapy (glucocorticoids, cytostatics, antibodies, drugs acting on immunophilins, interferon, tumor necrosis factor (TNF) binding proteins, mycophenolate) will not be included as these factors are known to possibly have an impact on the immune response to malgnancy.

## 8.7. Concomitant Interventions (treatments)

All concomitant and/or rescue treatments have to be recorded in the electronic CRF (eCRF), except for perioperative standard medication such as prophylactic antibiotics or heparin. Postoperative treatment against pain or PONV will be performed according to centers daily routine.

## 8.8. Study drug accountability

Investigational product supplies will be kept in a secure, limited access storage area under the recommended storage conditions.

The investigator will maintain accurate and adequate records including dates, lot number and doses given. Whenever available also total dosage of desflurane (ml) should be recorded.

## 8.9. Return or destruction of study drug / medical device

At completion of the study, there will be a final reconciliation of drug shipped, drug consumed, and drug remaining. This reconciliation will be logged on the drug accountability form, signed and dated. Any discrepancies noted will be investigated, resolved, and documented prior to return or destruction of unused study drug. Drug destroyed on site will be documented in the study files.

# STUDY ASSESSMENTS

## 9.1. Study flow chart of study procedures and assessments


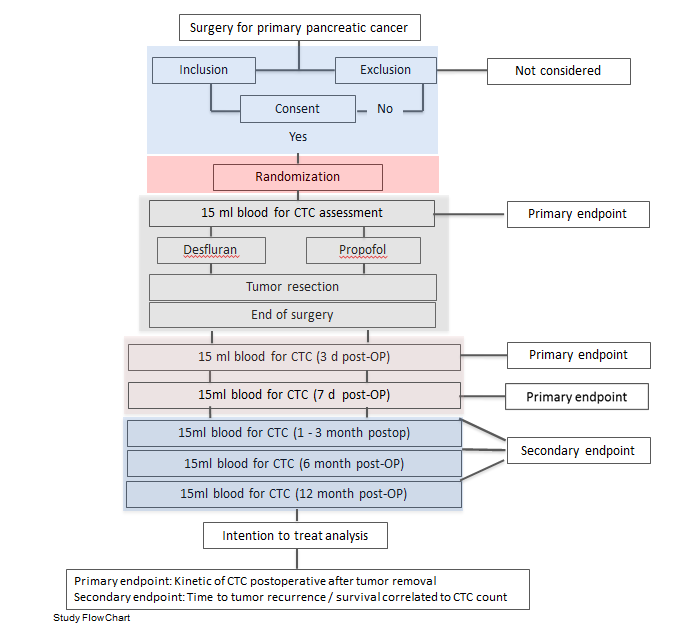


## 9.2. Assessments of outcomes

### Assessment of primary outcome

Blood samples will be collected before induction of general anesthesia (T0), at day 3 after surgery (T1), and at day 7 (T2).

The CTC detection procedure will be performed as described by the CellSearch® system manufacturer: 15 ml of whole blood will be aseptically collected into the CellSave preservative tubes and will be transported (at room temperature) to University Hospital Zurich, D LAB for subsequent analysis within 96 hours of blood collection.

Tubes for CTC cells detection will be kept in plastic bags specifically labeled with the visit number. In total 3 bags pro patient will be available: 1 for the attending anesthetist (visit 2) and 2 for the attending nursing staff on the ward (visit 3).

The blood samples will be eliminated after analysis.

### Assessment of secondary outcomes

CTC will be detected at 1-3 (prior chemo therapy), 6 and 12 months after surgery (T3, T4, T5). There will be 3 labeled CTC tubes available for the follow-up control of outpatients (visit at 1-3, 6 and 12 months).

Other secondary outcomes will be a) time to recurrence after primary surgery, b) overall survival after 12 month. To assess the secondary outcomes the CTC detection procedure will be performed and as well as a CTC scan will be conducted according to standards of the centers at 1- 3, 6- and/or 12-month follow-up visits, which are in compliance with the guidelines of European Society of Medical Oncology (ESMO)/European Society of Digestive Oncology (ESDO).

During the follow-up visits patient data will be recorded as described in **Table 3**.

### Assessment of safety outcomes

#### Adverse events (AE)

According to the Swiss Humanforschungsgesetz it is not necessary for category A trial to document abnormal findings as AE.

The following events will be considered to be recorded:

- if there is a reasonable possibility that a clinical outcome that deviated from the expected clinical course was caused by the investigational drug or study treatment (adverse reaction).
- if the clinical event meets the criteria for a SAE (i.e. death, life threatening, hospitalization, disability etc.)

#### Vital signs

Vital signs will be assessed as described in chapter 9.3

### Assessments in participants who prematurely stop the study

Subjects terminating the study (either regularly or prematurely) with

- reported ongoing SAE

will return for a follow-up investigation. This visit could take place up to 30 days after terminating the treatment period for the SAE. Follow-up information on the outcome will be recorded on the respective AE page in the eCRF. All other information has to be documented in the source documents. Source data have to be available upon request.

## 9.3. Procedures at each visit

| Perioperative data collection will be performed according to **Table 3**.  **Table 3** Data Collection |
| --- |
| Demographics |
| Date of visit |
| Inclusion/exclusion parameter |
| Eligibility |
| Randomization |
| Age, hight, length, weight |
| ASA classification |
| Concomitant therapy |
| Medical history |
| Current disease |
| Perioperativ Data |
| Date of surgery |
| Surgical intervention |
| Anesthesia duration (time of induction, end of application) |
| Regional anesthesia (y/n) total amount of medication, type of medication |
| Mean expiratory fraction in group desflurane (exp %) |
| Total amount of propofol in group propofol (mg)/total amount of desflurane if available (ml) |
| Mean core temperature (°C) |
| N° of units of blood transfusion |
| Pathology |
| Tumortype |
| Tumor classification TNM, grading |
| Resection (R0, R1) |
| Laboratory CTC |
| Date time line (T0 – T5) |
| Vital signs (heart rate, blood pressure, temperature) |
| Amount of CTC |
| Postoperative Admission |
| Hospital stay |
| Days on ICU/ PACU |
| Classifications of complications |
| Follow Up 1 - 3, 6, 12 month after primary intervention |
| Survival status (alive, dead), recording of death date if applicable |
| Recurrence y/n, recording of date of diagnosis of recurrence by imaging or biopsy or empiric clinical decision, location (local/systemic, if systemic: organ) |
| Re-operation (type) |
| Chemotherapy (y/n), medication, n° of cycles |
| Radiotherapy (y/n), n° of cycles |
| Checkbox ‘Lost of follow-up (attempted schedule for follow-up, telephone contact)’ |
| Casenote |
| Concomitant therapy |
| Medical history of concomitant medical disease |
| Study End Formular |

### Visit 1 (screening visit, preoperative phase)

- Information of patient and written informed consent
- Check for eligibility according to the inclusion and exclusion criteria
- Demographics, medical history, physical examination
- Concomitant therapy will be administered
- Potential confounders will be documented (pre-study assessment of bias)

### Visit 2 (day of surgery – before induction of general anesthesia,)

- Vital signs will be recorded, concomitant therapy will be administered
- Patient randomization to “group desflurane” (experimental) or to “group propofol” (control)
- 15ml of blood will be collected before induction of general anesthesia and sent for CTC cells assessment (T0)
- Study medication will be administered
- SAE will be recorded

### Visit 3 (day 3 after surgery)

- Vital signs will be recorded, concomitant therapy will be administered
- 15ml of blood will be collected and sent for CTC cells assessment around 8:00 AM, concomitant to routine postoperative laboratory tests (T1)
- SAE will be recorded

### Visit 4 (day 7 after surgery)

- Vital signs will be recorded, concomitant therapy will be administered
- 15ml of blood will be collected and sent for CTC cells assessment around 8:00 AM, concomitant to routine postoperative laboratory tests (T2)
- SAE will be recorded

### Visit 5 and 6 and 7 (pre-chemotherapy, 6- and 12-month follow-up)

- 15ml of blood will be collected and sent for CTC cells assessment (T3, T4, T5)
- Chemotherapy, radiotherapy will be recorded in the eCRF
- Tumor markers will be determined according to the practice of the study center
- Follow-up CT scans will be according to standard of the respective center (Guidelines of ESMO/ESDO)

# SAFETY

The Sponsor’s SOPs provide more detail on safety reporting.

During the entire duration of the study, all serious adverse events (SAEs) are collected and documented in source documents. Reportable events are recorded in the case report form (CRF). Study duration encompassed the time from when the participant signs the informed consent until the last protocol-specific procedure has been completed, including a safety follow-up period.

## 10.1. Definitions

**Adverse events**

Adverse events (AEs) are defined as any untoward medical occurrence in a patient or clinical investigation participant administered a pharmaceutical product and which does not necessarily have a causal relationship with this treatment. An AE can therefore be any unfavourable and unintended sign (including an abnormal laboratory finding), symptom, or disease temporally associated with the use of a medicinal study product, whether or not related to the medicinal study product. An AE may also consist of a new disease, an exacerbation of a pre-existing illness or condition, a recurrence of an intermittent illness or condition, a set of related signs or symptoms, or a single sign or symptom.

**Serious Adverse Event**

An SAE is any untoward medical occurrence that at any dose results in

• results in death,

• is life-threatening,

• requires participant hospitalization or prolongation of current hospitalization,

• results in persistent or significant disability/incapacity, or

• is a congenital anomaly/birth defect,

• any important medical event and any event which, though not included in the above, may jeopardise the participant or may require intervention to prevent one of the outcomes listed above.

Any other medically important condition that may be not immediately life-threatening or results in death or hospitalization but may jeopardize the participant or may require intervention to prevent one of the outcomes listed above should also usually (i.e. based on medical and scientific judgment) be considered serious.

**Unexpected Adverse Drug Reaction**

An “unexpected” adverse drug reaction is an adverse reaction, the nature or severity of which is not consistent with the applicable product information.

**Suspected unexpected serious adverse reaction (SUSAR)**

A serious adverse reaction, the nature or severity of which is suspected to be not consistent with the applicable product information.

**Safety Signals**

All suspected new risks and relevant new aspects of known adverse reactions that require safety-related measures.

## 10.2. Recording of Serious Adverse Events

Clinical investigators and ultimately the Principal Investigator (PI) in each center have the primary responsibility for SAE identification, documentation, grading, and assignment of attribution to the investigational agent/intervention.

All SAEs will be fully documented in the appropriate eCRF. For each SAE, the investigator will provide the onset, duration, intensity, treatment required, outcome and action taken with the investigational product.

The investigator assesses the causal relationship of each SAE according to the SAE reporting form.

## 10.3. Assessment of Serious Adverse Events

An unexpected SAE refers to any AE, the nature or severity of which is not consistent with the applicable product information.

The investigator will promptly review SAE to determine if the SAE meets the criteria for a suspected unexpected serious adverse reaction (SUSAR).

The assessment by the investigator with regard to the study drug relation is done according to the following definitions:

| Unrelated | - The event started in no temporal relationship to medicinal product applied and - The event can be definitely explained by underlying diseases or other situations. |
| --- | --- |
| Related | - The event started in a plausible temporal relationship to medicinal product applied and - The event cannot be definitely explained by underlying diseases or other situations. |

##

## 10.4. Reporting of Serious Adverse Events

The Investigator is responsible for reporting of any reportable SAE to the Sponsor **immediately**, at least within **24 hours**.

The Investigator is responsible for SAE reporting to the CEC according to the following details:

- - Reporting to CEC any SAE which resulted in death:

- **without delay**, and no later than **7 calendar days**.

- Reporting to CEC of fatal SAEs if evaluated as “suspected”, “unexpected” and “drug related” (SUSAR)

- **without delay** and no later than **7 calendar days** following awareness that event
 meets criteria for an SUSAR.

- Reporting to CEC of non-fatal SAEs if evaluated as “suspected”, “unexpected” and “drug related” (SUSAR):

**- promptly** and no later than **15 calendar days** following awareness that event
 meets criteria for a SUSAR.

The Sponsor is responsible for the reporting of unexpected ADRs to Swissmedic according to the normal pharmacovigilance practice.

All other SAEs will be summed up in the **annual safety report (ASR)**, containing:

- A summary of the safety profile of the drug studied as well as the safety issues that have arisen;
- A listing of all SUSARs that have occurred in Switzerland
- The accompanying letter provided with the Annual Safety Report should contain a short summary of the status of the clinical trial in Switzerland (number of centers open/closed, number of patients recruited/recruitment closed, and number of SADR/SUSAR.

The Sponsor is responsible for the reporting of unexpected ADRs to Swissmedic according to the normal pharmacovigilance practice.

Reporting of Safety Signals

All suspected new risks and relevant new aspects of known adverse reactions that require safety-related measures, i.e. so called safety signals, must be reported to the Sponsor within 24 hours. The Sponsor must report the safety signals within 7 days to the local Ethics Committee (local event via local Investigator).

In multicenter studies the following should be added:

The Sponsor must immediately inform all participating Investigators about all safety signals. The other in the trial involved Ethics Committees will be informed about safety signals in Switzerland via the Sponsor.

Reporting and Handling of Pregnancies

Pregnancy per se does not classify as an AE. However, AEs related to a pregnancy have to be reported like any other AEs. Pregnancy should be confirmed by a reliable laboratory test. In all studies not designed for pregnant participants or successful conception the following applies: Pregnant participants must be immediately withdrawn from the clinical study. All pregnancies occurring during the treatment phase of the study and within 30 days after discontinuation of study medication have to be reported to the Sponsor within 24 hours of the investigational sites knowledge of the pregnancy on the Initial Pregnancy Report Form. The Sponsor will contact the attendant physician by phone during pregnancy and after the estimated date of delivery to enquire about course and outcome of the *pregnancy. Course of the pregnancy and health status of the newborn child have to be documented on the Follow-Up Pregnancy Report Form*.

##

## 10.5. Follow up of (Serious) Adverse Events

Participants terminating the study (either regularly or prematurely) with

- reported ongoing SAE, or
- any ongoing AEs of laboratory values or of vital signs being beyond the alert limit

will return for a follow-up investigation. This visit will take place up to 30 days after terminating the treatment period. Follow-up information on the outcome will be recorded on the respective AE page in the eCRF. All other information has to be documented in the source documents. Source data has to be available upon request.

In case of participants lost to follow-up, efforts should be made and documented to contact the participant to encourage him/her to continue study participation as scheduled.

All new SAE or pregnancies that the investigators will be notified of within 30 days after discontinuation of study medication have to be reported in appropriate report forms and in the eCRF if required.

Follow-up investigations may also be necessary according to the investigator’s medical judgment even if the participant has no SAE at the end of the study. However, information related to these investigations does not have to be documented in the eCRF but must be noted in the source documents.

# STATISTICAL METHODS

## 11.1. Hypothesis

Our null hypothesis is that there is no difference in peak levels of CTC postoperatively between patients undergoing surgery for pancreatic cancer who receive desflurane or propofol. The alternative hypothesis is that there is a difference in peak levels of CTC in patients who receive desflurane versus patients who receive propofol.

## 11.2. Determination of sample size

We consider a ≥20% relative reduction in peak CTC count (day 3/day 7) in patients with the intervention as relevant compared to patients with the control intervention. A poisson regression of a dependent variable on a binomial distributed independent variable using a sample of 86 patients (both groups) will achieve 80% power at the 0.05 significance level for a 2-sided test to detect a response rate ratio of at least 0.77 for the intervention group compared with the control group count if the postoperative peak CTC rate was 6 and the mean exposure time was 1. The power calculation assumed 3 drop-outs per group.

## 11.3. Statistical criteria of termination of trial

We do not implement stopping rules for this very first trial since we do not expect differences between groups in terms of safety nor such a large effect of desflurane that would lead to a discontinuation of the trial.

## 11.4. Planned analyses

### Datasets to be analysed, analysis populations

We will base all analyses on the intention to treat principle where all patients are analyzed according to the random allocation.

### Primary Analysis

For the primary outcome, peak levels of CTC, we will use poisson regression analysis with CTC as the dependent variable and treatment allocation (desflurane vs. propofol) as independent variable and consider values smaller than 0.05 to be statistically significant. We will adjust the comparison for potential confounders if we find them to be distributed unevenly between groups (e.g. age, ASA, concomitant therapy, comorbidities and tumor pathology). The analysis will be performed by Prof. Milo Puhan and Dr. Julia Braun from the Epidemiology, Biostatistics and Prevention Institute of the University of Zurich.

### Secondary Analyses

For the analyses of the kinetics of postoperative CTC levels (from T0 to T2) We will use generalized linear mixed model with random effects to compare CTC levels between groups between baseline and up to 7 days postoperatively (T0 to T2), which takes the correlated structure of repeated measurements into consideration. To assess CTC levels at T3-5 we will also use poisson regression analysis as for the primary outcome. For the comparison of tumor recurrence (local recurrence or distant metastases) and overall survival between groups we will use Cox proportional hazards models. For all analyses, we will adjust the comparisons for potential confounders if we find them to be distributed unevenly between groups (e.g. age, ASA, concomitant therapy, comorbidities and tumor pathology). We do not intend to perform subgroup analyses. The analysis will be performed by Prof. Milo Puhan and Dr. Julia Braun from the Epidemiology, Biostatistics and Prevention Institute of the University of Zurich.

Full details of the statistical analysis plan will be published before the analysis of the trial.

### Interim analyses

Interim Analysis to check if assumptions about the peak CTC levels met has been done by the biostatistician team. This interim analysis showed that the sample sizes must to be correct (adding additional 30 patients based on the fact that the postoperative peak of CTC rate was 6 and not 10 as assumed at the beginning).

### Safety analysis

We will not perform interim safety analyses but follow all procedures described in Chapter 10 Safety.

### Deviation(s) from the original statistical plan

In case of changes to the statistical analyses plan we will describe the changes and the rationale for changes in the trial reports and publications.

## 11.5. Handling of missing data and drop-outs

As in earlier and similar trials we do not expect much missing data (e.g. >5% for a variable) nor many drop outs so we plan to perform complete case analyses.

# Eligibility of the Project Site(s)

This trial will be conducted in 3 Swiss tertiary care centers Kantonsspital Winterthur, Kantonsspital St. Gallen and Stadtspital Triemli. These hospitals provide ideal conditions for clinical research (dedicated study nurses, research-orientated infrastructures and high standards of quality control). Beyond this, considerable efforts will be made to ensure high protocol compliance; (e.g. regular meetings with the anesthesia team to identify potential enrollment or organizational issues).

# QUALITY ASSURANCE AND CONTROL

The Sponsor is implementing and maintaining quality assurance and quality control systems with written SOPs and Working Instructions to ensure that trials are conducted and data are generated, documented (record), and reported in compliance with the protocol, GCP, and applicable regulatory requirement(s).

Monitoring and audits will be conducted by the chief study nurse (Sabine Kern) during the course of the study for quality assurance purposes.

## 13.1. Data handling and record keeping / archiving

The study will strictly follow the protocol. If any changes become necessary, they must be laid down in an amendment to the protocol. All amendments of the protocol must be signed by the Sponsor and submitted to IEC.

### 13.1.1. Case Report Forms

The investigators will use eCRF, one for each enrolled study participant, to be filled in with all relevant data pertaining to the subject during the study. All subjects who either entered the study or were considered not eligible or were eligible but not enrolled into the study additionally have to be documented on a screening log in each center. The Principal Investigator will document the participation of each study subject on the Enrollment Log.

For data and query management, monitoring, reporting and coding an internet-based secure data base secuTrial® developed in agreement to the GCP guidelines provided by the Clinical Trials Center (CTC) Zurich will be used for this study. It is the responsibility of the investigator to assure that all data in the course of the study will be entered completely and correctly in the respective database. Corrections in the eCRF can only be done by study investigators or authorized members of the research group. In case of corrections the original data entries will be archived in the system and can be made visible. For all data entries and corrections date, time of day and person who is performing the entries will be generated automatically*.*

eCRFs must be kept current to reflect subject status at each phase during the course of study. Subjects must not to be identified in the eCRF by name. Appropriate coded identification (e.g. Subject Number) must be used.

It must be assured that any authorized person, who may perform data entries and changes in the eCRF, can be identified. A list with signatures and initials of all authorized persons will be filed in the study site file and the trial master file, respectively.

Documented medical histories and narrative statements relative to the subject's progress during the study will be maintained. These records will also include the following: originals or copies of laboratory and other medical test results must be kept on file with the individual subject's eCRF.

The investigators assure to perform a complete and accurate documentation of the subject data in the eCRF. All data entered into the eCRF must also be available in the individu­al subject file either as print-outs or as notes taken by either the investigator or another responsible person assigned by the investigator.

Any patient files and source data must be archived for the longest possible period of time according to the feasibility of the investigational site, e.g. hospital, institution or private practice.

### Specification of source documents

The following documents are considered source data, including but not limited to:

- SAE worksheets
- Nurse records, records of clinical coordinators, and
- Medical records from other department(s), or other hospital(s), or discharge letters and correspondence with other departments/hospitals, if subject visited any during the study period and the post study period.

Source data must be available at the site to document the existence of the study subjects and substantiate the integrity of study data collected. Source data must include the original documents relating to the study, as well as the medical treatment and medical history of the subject.

The following information (at least but not limited to) should be included in the source documents:

• Demographic data (age, sex)

• Inclusion and exclusion criteria details

• Participation in study and signed and dated informed consent forms

• Visit dates

• Medical history and physical examination details (only pathological findings)

• Key efficacy and safety data (as specified in the protocol)

• Results of relevant examinations

• Laboratory printouts

• Dispensing and return of study drug details

• Reason for premature discontinuation

• Randomization number

### 13.1.2 Randomization number Record keeping / archiving

Logistic access to randomization will be provided by secuTrial® system. Randomization key will be locked secure in the TMF.

Essential documents must be retained for at least 10 years after the regular end or a premature termination of the respective study (KlinV Art. 25).

## 13.2. Data management

The study will strictly follow the protocol. If any changes become necessary, they must be laid down in an amendment to the protocol. All amendments of the protocol must be signed by the Sponsor and submitted to IEC.

### Data management system

For data and query management, monitoring, reporting and coding an internet-based secure data base secuTrial® developed in agreement to the GCP guidelines provided by the Clinical Trials Center (CTC) Zurich will be used for this study.

It is the responsibility of the investigator to assure that all data in the course of the study will be entered completely and correctly in the respective database. Corrections in the eCRF may only be done by the investigator or by other authorized persons. In case of corrections the original data entries will be archived in the system and can be made visible. For all data entries and corrections date, time of day and person who is performing the entries will be generated automatically*.*

The investigators will use eCRF, one for each enrolled study participant, to be filled in with all relevant data pertaining to the subject during the study. All subjects who either entered the study or were considered not eligible or were eligible but not enrolled into the study additionally have to be documented on a screening log. The investigator will document the participation of each study subject on the Enrollment Log.

### Data security, access and back-up

Data generation, transmission, archiving and analysis of personal data within this project, strictly follows Swiss legal requirements for data protection.

Access to non – anonymized data is strictly limited by:

Sponsor, Principal – investigator(s), Co – Investigator(s) and study nurse(s).

### Analysis and archiving

Data Analysis will be performed after enrollment of the last patient and completion of the study. No interim analysis are planned

Essential documents must be retained for at least 10 years after the regular end or a premature termination of the respective study.

Any patient files and source data must be archived for the longest possible period of time according to the feasibility of the investigational site.

### Electronic and central data validation

Describe how data are validated.

For data and query management, monitoring, reporting and coding an internet-based secure data base secuTrial® developed in agreement to the GCP guidelines provided by the Clinical Trials Unit (CTU) Zurich will be used for this study. It is the responsibility of the investigator to assure that all data in the course of the study will be entered completely and correctly in the respective database. Corrections in the eCRF may only be done by the investigator or by other authorized persons. In case of corrections the original data entries will be archived in the system and can be made visible. For all data entries and corrections date, time of day and person who is performing the entries will be generated automatically*.*

eCRFs must be kept current to reflect subject status at each phase during the course of study. Subjects must not to be identified in the eCRF by name. Appropriate coded identification (e.g. Subject Number) must be used.

It must be assured that any authorized person, who may perform data entries and changes in the eCRF, can be identified. A list with signatures and initials of all authorized persons will be filed in the study site file and the trial master file, respectively.

## 13.3. Monitoring

The Sponsor site will provide monitoring, which is performed by the chief study nurse. The extent and nature of monitoring activities based on the objective and design of the study will be defined in a study specific Monitoring Plan.

All original data including all patient files, progress notes and copies of laboratory and medical test results must be available for monitoring. The monitor will review all or a part of the eCRFs and written informed consents. The accuracy of the data will be verified by reviewing the above referenced documents.

## 13.4. Audits and inspections

A quality assurance audit/inspection of this study may be conducted by the competent authority or CEC, respectively. The quality assurance auditor/inspector will have access to all medical records, the investigator's study related files and correspondence, and the informed consent documentation that is relevant to this clinical study.

The investigator will allow the persons being responsible for the audit or the inspection to have access to the source data/documents and to answer any questions arising. All involved parties will keep the patient data strictly confidential.

## 13.5. Confidentiality, data protection

Direct access to source documents will be permitted for purposes of monitoring, audits and inspections

The investigator will allow the persons being responsible for the audit or the inspection to have access to the source data/documents and to answer any questions arising. All involved parties will keep the patient data strictly confidential.

## 13.6. Storage of biological material and related health data

N/A

# PUBLICATION AND DISSEMINATION POLICY

After the statistical analysis of this trial the sponsor will make every endeavour to publish the data in a medical journal

# FUNDING AND SUPPORT

## 15.1. Funding

Baxter AG provided CHF 20000 grant money to be able to initiate the trial. The company is neither involved into the study design nor data collection.

## 15.2. Other support

The research team of the Sponsor is involved in supporting the proper flow of the study, particularly with regard to determination of CTC.

# INSURANCE

Insurance is covered by “Versicherung für klinische Versuche und nichtklinische Versuche“ by Zürich Versicherungs-Gesellschaft AG (Policy no 15.369.591).

Any damage developed in relation to study participation is covered by this insurance. So as not to forfeit their insurance cover, the participants themselves must strictly follow the instructions of the study personnel. Participants must not be involved in any other medical treatment without permission of the principal investigator (emergency excluded). Medical emergency treatment must be reported immediately to the investigator. The investigator must also be informed instantly, in the event of health problems or other damages during or after the course of study treatment.

The investigator will allow delegates of the insurance company to have access to the source data/documents as necessary to clarify a case of damage related to study participation. All involved parties will keep the patient data strictly confidential.

A copy of the insurance certificate will be placed in the Investigator’s Site File.

# REFERENCES

1. Siegel R, Naishadham D, Jemal A. Cancer statistics, 2013. *CA Cancer J Clin* 2013;**63**(1): 11-30.

2. Hidalgo M. Pancreatic cancer. *N Engl J Med* 2010;**362**(17): 1605-1617.

3. Raimondi S, Maisonneuve P, Lowenfels AB. Epidemiology of pancreatic cancer: an overview. *Nat Rev Gastroenterol Hepatol* 2009;**6**(12): 699-708.

4. Arvold ND, Ryan DP, Niemierko A, Blaszkowsky LS, Kwak EL, Wo JY, Allen JN, Clark JW, Wadlow RC, Zhu AX, Fernandez-Del Castillo C, Hong TS. Long-term outcomes of neoadjuvant chemotherapy before chemoradiation for locally advanced pancreatic cancer. *Cancer* 2012;**118**(12): 3026-3035.

5. Cristofanilli M, Budd GT, Ellis MJ, Stopeck A, Matera J, Miller MC, Reuben JM, Doyle GV, Allard WJ, Terstappen LW, Hayes DF. Circulating tumor cells, disease progression, and survival in metastatic breast cancer. *N Engl J Med* 2004;**351**(8): 781-791.

6. Hayes DF, Cristofanilli M, Budd GT, Ellis MJ, Stopeck A, Miller MC, Matera J, Allard WJ, Doyle GV, Terstappen LW. Circulating tumor cells at each follow-up time point during therapy of metastatic breast cancer patients predict progression-free and overall survival. *Clin Cancer Res* 2006;**12**(14 Pt 1): 4218-4224.

7. Zhang L, Riethdorf S, Wu G, Wang T, Yang K, Peng G, Liu J, Pantel K. Meta-analysis of the prognostic value of circulating tumor cells in breast cancer. *Clin Cancer Res* 2012;**18**(20): 5701-5710.

8. Tjensvoll K, Nordgard O, Smaaland R. Circulating tumor cells in pancreatic cancer patients: methods of detection and clinical implications. *International journal of cancer Journal international du cancer* 2014;**134**(1): 1-8.

9. Allard WJ, Matera J, Miller MC, Repollet M, Connelly MC, Rao C, Tibbe AG, Uhr JW, Terstappen LW. Tumor cells circulate in the peripheral blood of all major carcinomas but not in healthy subjects or patients with nonmalignant diseases. *Clin Cancer Res* 2004;**10**(20): 6897-6904.

10. Faltas B. Cornering metastases: therapeutic targeting of circulating tumor cells and stem cells. *Front Oncol* 2012;**2**: 68.

11. Gall TM, Jacob J, Frampton AE, Krell J, Kyriakides C, Castellano L, Stebbing J, Jiao LR. Reduced Dissemination of Circulating Tumor Cells With No-Touch Isolation Surgical Technique in Patients With Pancreatic Cancer. *JAMA surgery* 2014.

12. Kurosawa S. Anesthesia in patients with cancer disorders. *Curr Opin Anaesthesiol* 2012;**25**(3): 376-384.

13. Fukui K, Werner C, Pestel G. [Influence of anesthesia procedure on malignant tumor outcome]. *Anaesthesist* 2012;**61**(3): 193-201.

14. Gottschalk A, Sharma S, Ford J, Durieux ME, Tiouririne M. Review article: the role of the perioperative period in recurrence after cancer surgery. *Anesth Analg* 2010;**110**(6): 1636-1643.

15. Tavare AN, Perry NJ, Benzonana LL, Takata M, Ma D. Cancer recurrence after surgery: direct and indirect effects of anesthetic agents. *International journal of cancer Journal international du cancer* 2011;**130**(6): 1237-1250.

16. Muller-Edenborn B, Roth-Z'graggen B, Bartnicka K, Borgeat A, Hoos A, Borsig L, Beck-Schimmer B. Volatile anesthetics reduce invasion of colorectal cancer cells through down-regulation of matrix metalloproteinase-9. *Anesthesiology* 2012;**117**(2): 293-301.

17. Lurje G, Schiesser M, Claudius A, Schneider PM. Circulating tumor cells in gastrointestinal malignancies: current techniques and clinical implications. *J Oncol* 2010;**2010**: 392652.

# APPENDICES

Appendix 1: Fachinformation Desfluran

**Supplementary Figures and Tables**

**Figure S1**

**
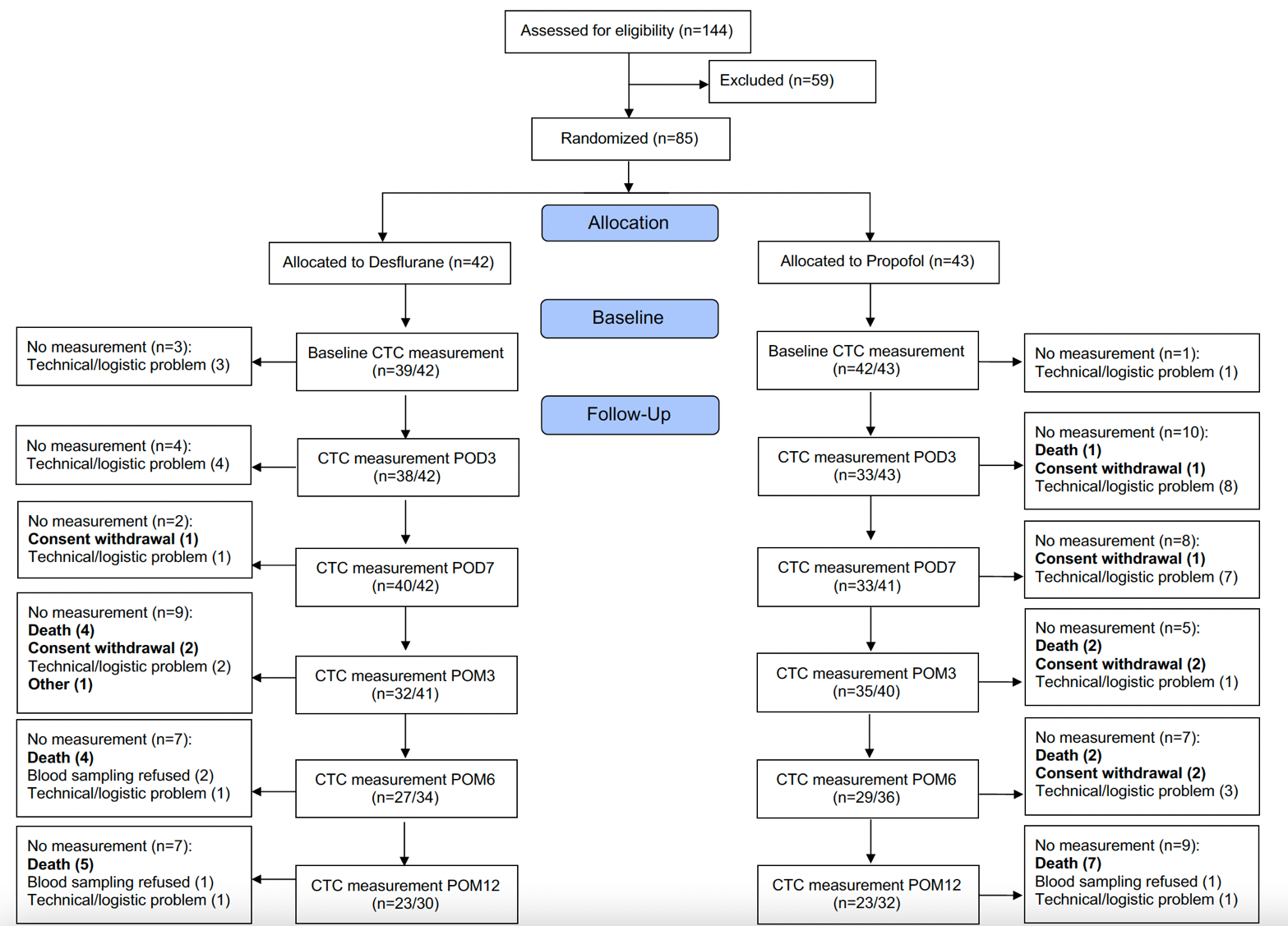
**

**Figure S1:** Trial flow diagram. Drop-outs and measurement errors at the different levels of the study are indicated.

Abbreviations: circulating tumor cells (CTC), postoperative day (POD), postoperative months (POM)

**Figure S2**

**
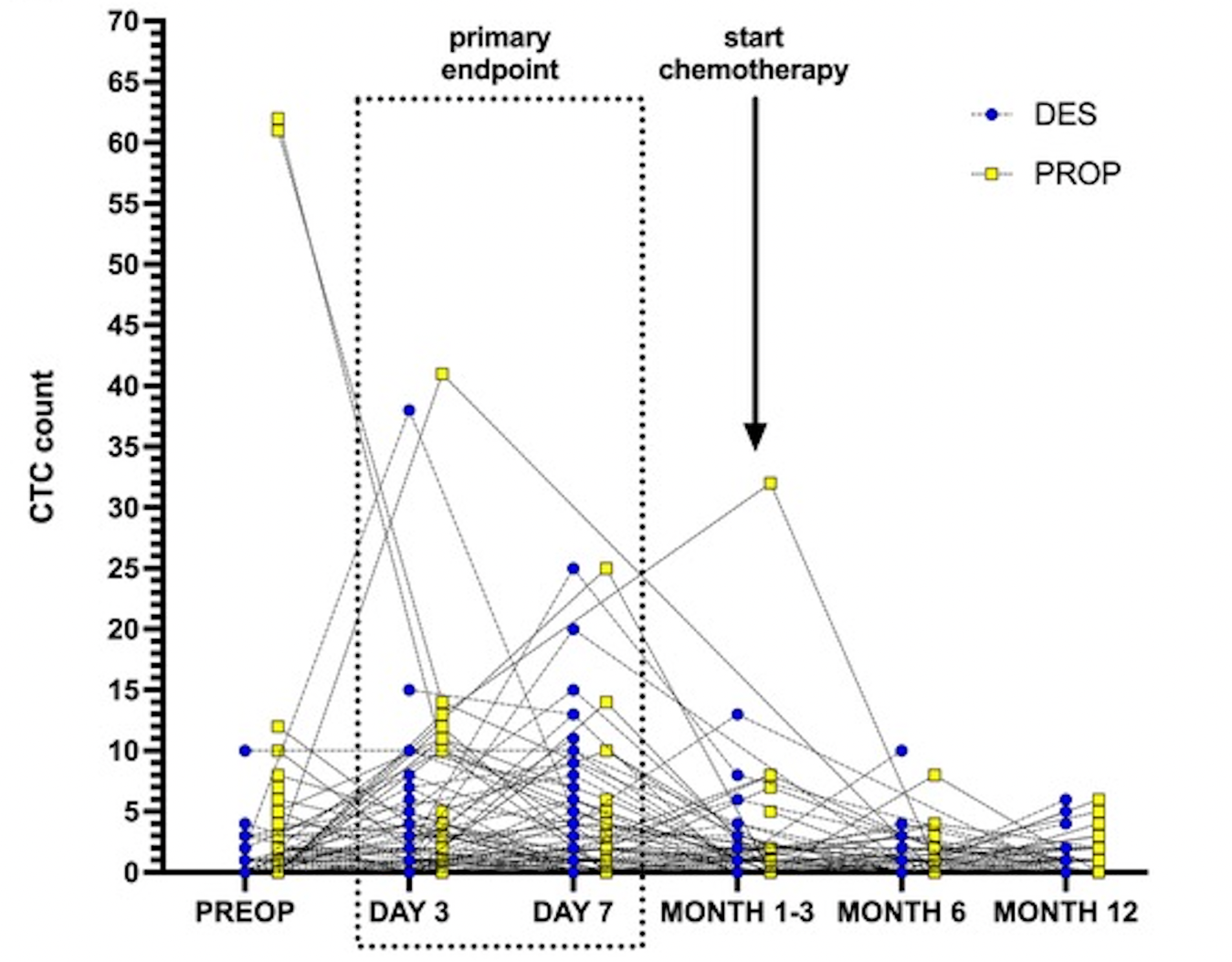
**

**Figure S2:** Circulating tumor cell measurements over the entire study duration and patient trajectories.

Abbreviations: circulating tumor cells (CTC), desflurane (DES), propofol (PROP)

**Figure S3A Figure S3B**


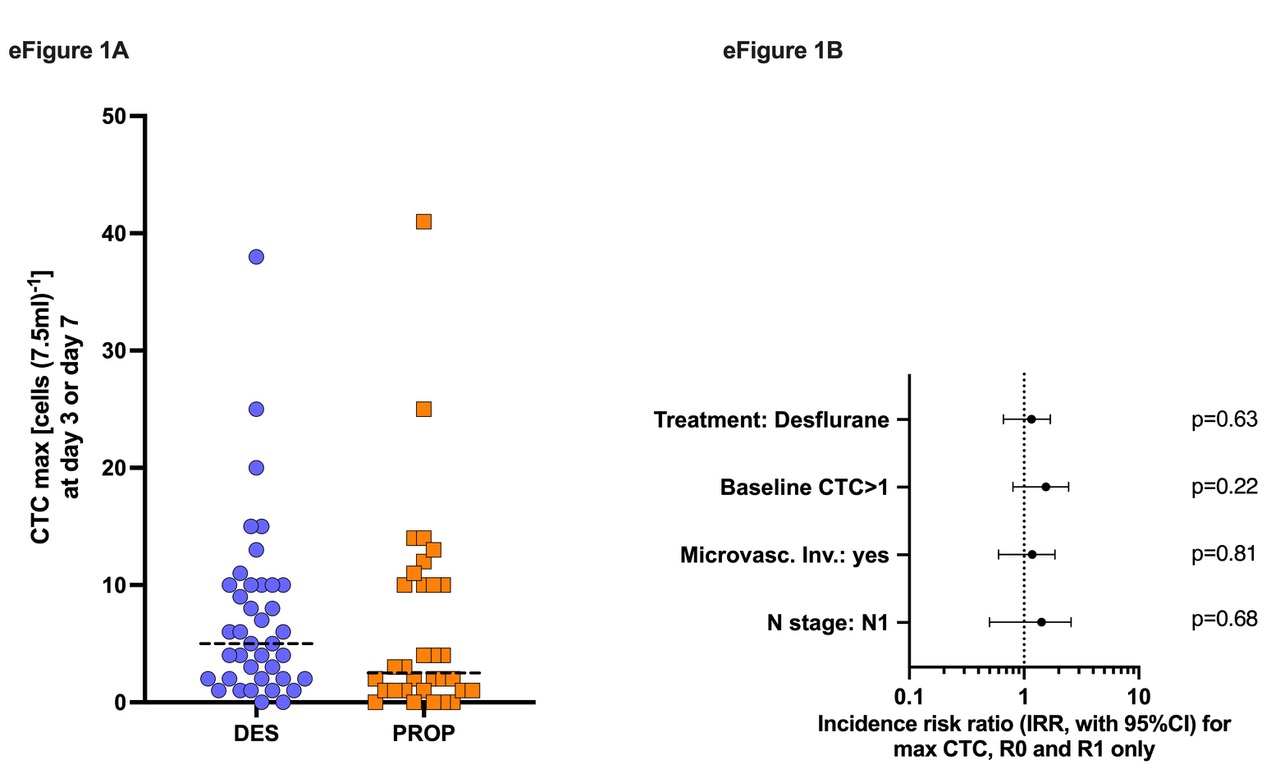


**Figure S3**: Subgroup analysis: Maximum circulating tumor cell values in the two treatment groups (**A**) of patients undergoing (macroscopic) curative resection. Results of a negative binomial regression model considering the confounders group allocation, baseline CTC>1, microvascular invasion, and lymph node stage (N) (**B**).

Abbreviations: circulating tumor cells (CTC). desflurane (DES), propofol (PROP), lymph node (N), incidence risk ratio (IRR), 95% confidence interval (CI)

**Figure S4A Figure S4B**

**
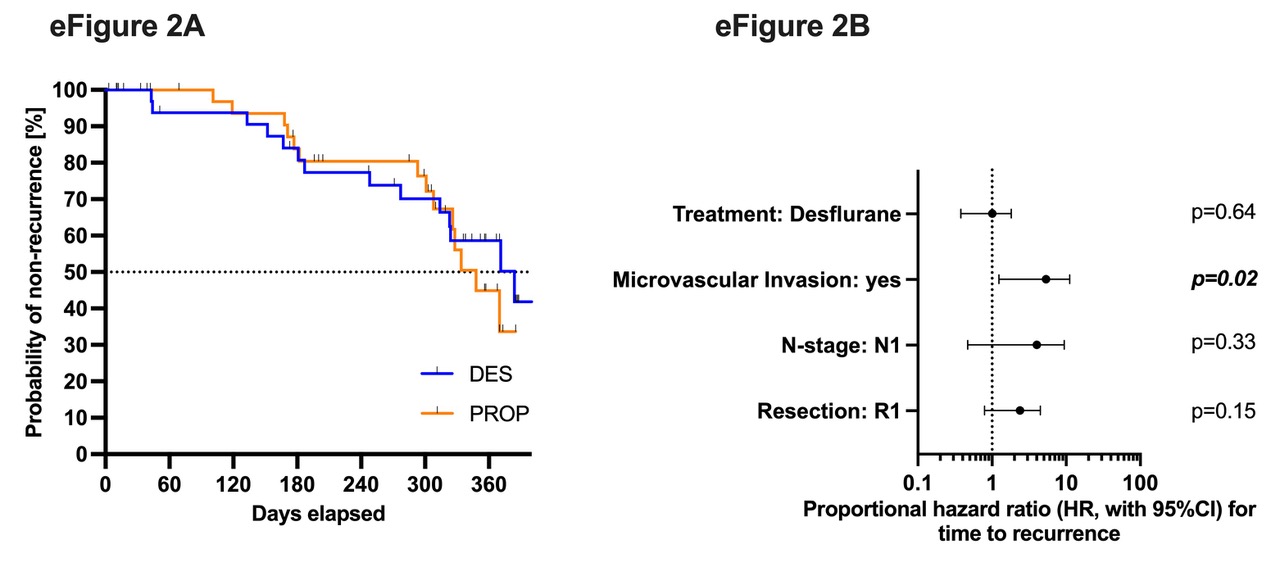
**

**Figure S4**: Subgroup analysis: Probability of non-recurrence as shown by Kaplan-Meier in patients undergoing (macroscopic) curative resection (**A**). COX regression model for the time to recurrence including the confounders group allocation, microvascular invasion, lymph node stage (N1), and resection (R1) (**B**).

Abbreviations: desflurane (DES), propofol (PROP), hazard ratio (HR), 95% confidence interval (95%CI)

**Figure S5A Figure S5B**

**
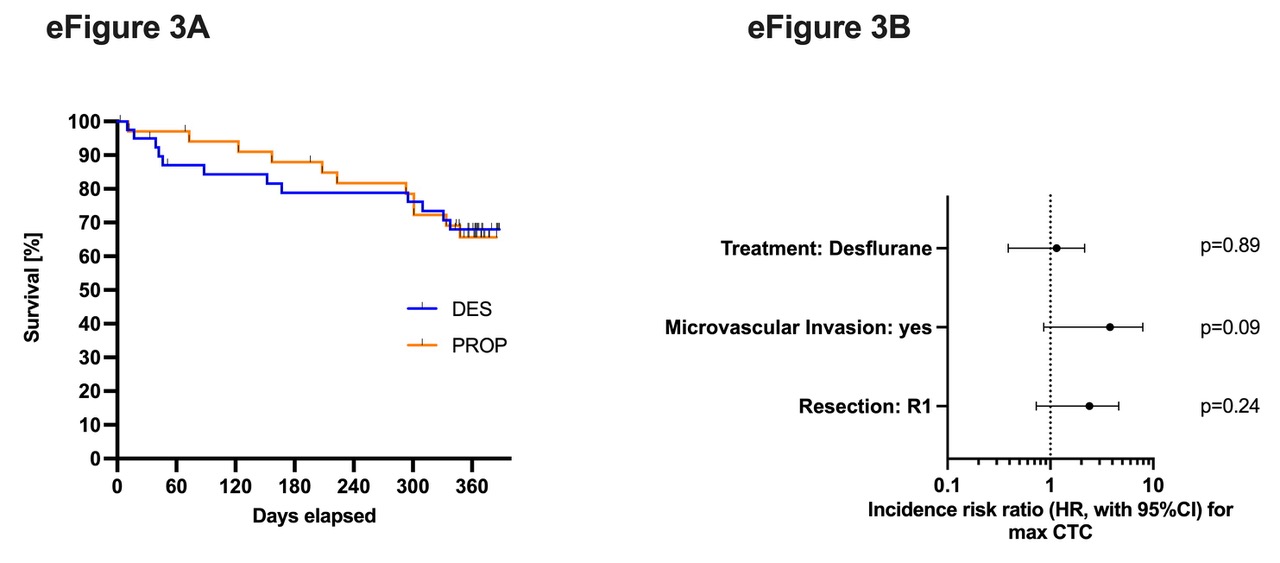
**

**Figure S5**: Subgroup analysis: Probability of survival as shown by Kaplan-Meier in patients undergoing (macroscopic) curative resection (**A**). COX regression model for the time to death and considering the confounding factors treatment, microvascular invasion, and resection (R1) (**B**).

Abbreviations: desflurane (DES), propofol (PROP), hazard ratio (HR), 95% confidence interval (95%CI), circulating tumor cells (CTCs).

**Figure S6**

**Figure S6**: Maximum circulating tumor cell values of all patients (pooled) in the two treatment groups of patients undergoing (macroscopic) curative resection in order to determine the impact of surgery on circulating tumor cell count.

Abbreviations: circulating tumor cells (CTCs).

| **Table S1: Patient and Tumor Characteristics** |  |  |
| --- | --- | --- |
| **Variable** | **Desflurane** | **Propofol** |
|  |  |  |
| Patient characteristics | **n = 42** | **n = 43** |
| Age (y), median (IQR) | 71 (63-79) | 71 (65-75) |
| Sex - female/male, n | 17/25 (40/60%) | 25/18 (51/49%) |
| Charlson Comorbidity Index, median (IQR) | 2 (0-3) | 2 (1-3) |
| BMI (kg/m^2^), median (IQR) | 24.9 (22.1-29.2) | 25.8 (22.7-27.4) |
| ASA-class number 1/2/3, n | 4/12/26 (10/29/62%) | 0/15/28 (0/35/65%) |
| Centre 1/2/3, n | 15/12/15 (36/29/36%) | 15/13/15 (35/30/35%) |
| Biopsy proven adenocarcinoma prior to surgery, n (%) | 23 (55%) | 26 (60%) |
| Biliary stent preoperative, n (%) | 15 (37%) | 13 (30%) |
| Creatinine baseline (µmol/L), median (IQR) | 70 (58-86) | 68 (62-77) |
| Bilirubin baseline (µmol/L), median (IQR) | 37 (12-127) | 16(10-114) |
| INR baseline, median (IQR) | 1.0 (0.9-1.1) | 1.0 (0.9-1.1) |
| Hb (g/l), median (IQR) | 131 (113-141) | 129 (123-142) |
| CA 19-9, median (IQR) | 206 (61-1430) | 137 (16-476) |
| CEA, median (IQR) | 3 (2-6) | 3 (2-6) |
| Tumor characteristics |  |  |
| Location in preop imaging |  |  |
| pancreatic head, n | 36 (85%) | 33 (77%) |
| pancreatic corpus, n | 4 (10%) | 6 (14%) |
| pancreatic tail, n | 2 (5%) | 4 (9%) |
| Completeness of resection | **n=40** | **n=34** |
| RO, n | 32 (80%) | 28 (82%) |
| R1, n | 8 (20%) | 6 (18%) |
| R2, n | 0 (0%) | 0 |
| Curative resection not possible/ not performed, n (%) | 2 (5%) | 9 (21%) |
| Pathology (available only in patients resected) | **n=40** | **n=34** |
| Grade of differentiation G1/G2/G3 (denominator) | 1/15/24 (40) | 0/15/16 (34) |
| Microvascular invasion, no/yes (denominator) | 15/25 (40) | 13/21 (34) |
| Macrovascular invasion no/yes (denominator) | 27/13 (40) | 26/7 (33) |
| pT staging (n) T0/T1/T2/ T3/T4/Tx (denominator) | 0/0/18/21/1/0 (40) | 0/2/14/15/1/2 (34) |
| pN staging (n) N0/N1/Nx (denominator) | 3/36/1 (40) | 8/26/0 (34) |
| pM staging (n) M0/M1/Mx (denominator) | 36/4/0 (40) | 32/2/0 (34) |

Data are presented as median and interquartile range (IQR) or as absolute numbers (n) and percentage (%). Abbreviations: y: years; kg: kilogram; m^2^: square meters; µmol/L: micromole per liter; INR: international normalized ratio; CA 19-9: carbohydrate antigen 19-9; CEA: carcinoembryonic antigen; UICC: union for international cancer control; R: residual; pT: pathology staging of tumor; pN: pathology staging of lymph nodes (TNM UICC classification version 7)

| **Table S2: Procedure characteristics and clinical outcomes** | | | |
| --- | --- | --- | --- |
| **Variate** | **Desflurane** | **Propofol** |  |
|  |  |  |  |
| **Surgical data** | **n=42** | **n=43** |  |
| Type of resection, n |  |  |  |
| Whipple resection | 31 | 25 |  |
| Classic | 16 | 16 |  |
| Duodenal sparing | 14 | 9 |  |
| Pylorus-preserving | 1 | 0 |  |
| Distal pancreatectomy | 5 | 6 |  |
| Total pancreatectomy | 4 | 2 |  |
| Central pancreatectomy | 0 | 1 |  |
| Aborted resection/palliative | 2 | 9 |  |
| Laparoscopic approach, n | 3 (7%) | 5 (12%) |  |
| **Procedure details** |  |  |  |
| Duration of procedure (min) | 432±132 | 369±145 |  |
| Blood loss (mL) | 402±220 | 307±247 |  |
| **Anesthesia details** |  |  |  |
| Regional anesthesia, yes, n | 41 (98%) | 41 (95%) |  |
| Perioperative opioid therapy, n | 43 (100%) | 43 (100%) |  |
| Perioperative steroid therapy, n | 15 (36%) | 10 (23%) |  |
| Body temperature | 36.8±0.5 | 36.5±0.5 |  |
| Crystalloids (mL) | 3855±1559 | 3030±2135 |  |
| **Clinical outcome** |  |  |  |
| Any complication during hospitalization, n (denominator) | 27 (41) | 29 (41) |  |
| Complication Dindo-Clavien I and II | 13 | 20 |  |
| Complication Dindo-Clavien ≥ IIIA | 15 | 11 |  |
| Complication Dindo-Clavien ≥ IIIB | 8 | 8 |  |
| Complication Dindo-Clavien = V (mortality) | 4 | 2 |  |
| CCI, median (IQR) | 23 (0-34) | 21 (9-31) |  |
| Post-pancreatectomy fistula | 13 | 14 |  |
| 90-day mortality n/denominator | 5/42 (12%) | 2/42 (5%) |  |
| Length of ICU stay, median (IQR) | 2 (1-3) | 2 (1-5) |  |
| Length of hospital stay, median (IQR) | 18 (14-26) | 14 (11-20) |  |
| **Adjuvant chemotherapy (3 months postoperatively)** |  |  |  |
| No | 7 | 9 |  |
| Yes | 23 | 20 |  |
| Deceased before initiation of chemotherapy | 7 | 4 |  |
| Consent withdrawn | 3 | 1 |  |
| Not resectable | 2 | 9 |  |

Data are presented as median and interquartile range (IQR), as mean ± standard deviation (SD), or as absolute numbers (n) and percentage (%). Abbreviations: min: minutes; mL: millilitres; CCI: Charlson comorbidity index
